# A global metagenomic map of urban microbiomes and antimicrobial resistance

**DOI:** 10.1016/j.cell.2021.05.002

**Published:** 2021-06-24

**Authors:** David Danko, Daniela Bezdan, Evan E. Afshin, Sofia Ahsanuddin, Chandrima Bhattacharya, Daniel J. Butler, Kern Rei Chng, Daisy Donnellan, Jochen Hecht, Katelyn Jackson, Katerina Kuchin, Mikhail Karasikov, Abigail Lyons, Lauren Mak, Dmitry Meleshko, Harun Mustafa, Beth Mutai, Russell Y. Neches, Amanda Ng, Olga Nikolayeva, Tatyana Nikolayeva, Eileen Png, Krista A. Ryon, Jorge L. Sanchez, Heba Shaaban, Maria A. Sierra, Dominique Thomas, Ben Young, Omar O. Abudayyeh, Josue Alicea, Malay Bhattacharyya, Ran Blekhman, Eduardo Castro-Nallar, Ana M. Cañas, Aspassia D. Chatziefthimiou, Robert W. Crawford, Francesca De Filippis, Youping Deng, Christelle Desnues, Emmanuel Dias-Neto, Marius Dybwad, Eran Elhaik, Danilo Ercolini, Alina Frolova, Dennis Gankin, Jonathan S. Gootenberg, Alexandra B. Graf, David C. Green, Iman Hajirasouliha, Jaden J.A. Hastings, Mark Hernandez, Gregorio Iraola, Soojin Jang, Andre Kahles, Frank J. Kelly, Kaymisha Knights, Nikos C. Kyrpides, Paweł P. Łabaj, Patrick K.H. Lee, Marcus H.Y. Leung, Per O. Ljungdahl, Gabriella Mason-Buck, Ken McGrath, Cem Meydan, Emmanuel F. Mongodin, Milton Ozorio Moraes, Niranjan Nagarajan, Marina Nieto-Caballero, Houtan Noushmehr, Manuela Oliveira, Stephan Ossowski, Olayinka O. Osuolale, Orhan Özcan, David Paez-Espino, Nicolás Rascovan, Hugues Richard, Gunnar Rätsch, Lynn M. Schriml, Torsten Semmler, Osman U. Sezerman, Leming Shi, Tieliu Shi, Rania Siam, Le Huu Song, Haruo Suzuki, Denise Syndercombe Court, Scott W. Tighe, Xinzhao Tong, Klas I. Udekwu, Juan A. Ugalde, Brandon Valentine, Dimitar I. Vassilev, Elena M. Vayndorf, Thirumalaisamy P. Velavan, Jun Wu, María M. Zambrano, Jifeng Zhu, Sibo Zhu, Christopher E. Mason, The International MetaSUB Consortium, Natasha Abdullah, Marcos Abraao, Ait-hamlat Adel, Muhammad Afaq, Faisal S. Al-Quaddoomi, Ireen Alam, Gabriela E. Albuquerque, Alex Alexiev, Kalyn Ali, Lucia E. Alvarado-Arnez, Sarh Aly, Jennifer Amachee, Maria G. Amorim, Majelia Ampadu, Muhammad Al-Fath Amran, Nala An, Watson Andrew, Harilanto Andrianjakarivony, Michael Angelov, Verónica Antelo, Catharine Aquino, Álvaro Aranguren, Luiza F. Araujo, Hitler Francois Vasquez Arevalo, Jenny Arevalo, Carme Arnan, Lucia Elena Alvarado Arnez, Fernanda Arredondo, Matthew Arthur, Freddy Asenjo, Thomas Saw Aung, Juliette Auvinet, Nuria Aventin, Sadaf Ayaz, Silva Baburyan, Abd-Manaaf Bakere, Katrin Bakhl, Thais F. Bartelli, Erdenetsetseg Batdelger, François Baudon, Kevin Becher, Carla Bello, Médine Benchouaia, Hannah Benisty, Anne-Sophie Benoiston, Joseph Benson, Diego Benítez, Juliana Bernardes, Denis Bertrand, Silvia Beurmann, Tristan Bitard-Feildel, Lucie Bittner, Christina Black, Guillaume Blanc, Brittany Blyther, Toni Bode, Julia Boeri, Bazartseren Boldgiv, Kevin Bolzli, Alexia Bordigoni, Ciro Borrelli, Sonia Bouchard, Jean-Pierre Bouly, Alicia Boyd, Gabriela P. Branco, Alessandra Breschi, Björn Brindefalk, Christian Brion, Alan Briones, Paulina Buczansla, Catherine M. Burke, Aszia Burrell, Alina Butova, Irvind Buttar, Jalia Bynoe, Sven Bönigk, Kari O. Bøifot, Hiram Caballero, Xiao Wen Cai, Dayana Calderon, Angela Cantillo, Miguel Carbajo, Alessandra Carbone, Anais Cardenas, Katerine Carrillo, Laurie Casalot, Sofia Castro, Ana V. Castro, Astred Castro, Ana Valeria B. Castro, Simone Cawthorne, Jonathan Cedillo, Salama Chaker, Jasna Chalangal, Allison Chan, Anastasia I. Chasapi, Starr Chatziefthimiou, Sreya Ray Chaudhuri, Akash Keluth Chavan, Francisco Chavez, Gregory Chem, Xiaoqing Chen, Michelle Chen, Jenn-Wei Chen, Ariel Chernomoretz, Allaeddine Chettouh, Daisy Cheung, Diana Chicas, Shirley Chiu, Hira Choudhry, Carl Chrispin, Kianna Ciaramella, Erika Cifuentes, Jake Cohen, David A. Coil, Sylvie Collin, Colleen Conger, Romain Conte, Flavia Corsi, Cecilia N. Cossio, Ana F. Costa, Delisia Cuebas, Bruno D’Alessandro, Katherine E. Dahlhausen, Aaron E. Darling, Pujita Das, Lucinda B. Davenport, Laurent David, Natalie R. Davidson, Gargi Dayama, Stéphane Delmas, Chris K. Deng, Chloé Dequeker, Alexandre Desert, Monika Devi, Felipe S. Dezem, Clara N. Dias, Timothy Ryan Donahoe, Sonia Dorado, LaShonda Dorsey, Valeriia Dotsenko, Steven Du, Alexandra Dutan, Naya Eady, Jonathan A. Eisen, Miar Elaskandrany, Lennard Epping, Juan P. Escalera-Antezana, Cassie L. Ettinger, Iqra Faiz, Luice Fan, Nadine Farhat, Emile Faure, Fazlina Fauzi, Charlie Feigin, Skye Felice, Laís Pereira Ferreira, Gabriel Figueroa, Aubin Fleiss, Denisse Flores, Jhovana L. Velasco Flores, Marcos A.S. Fonseca, Jonathan Foox, Juan Carlos Forero, Aaishah Francis, Kelly French, Pablo Fresia, Jacob Friedman, Jaime J. Fuentes, Josephine Galipon, Mathilde Garcia, Laura Garcia, Catalina García, Annie Geiger, Samuel M. Gerner, Sonia L. Ghose, Dao Phuong Giang, Matías Giménez, Donato Giovannelli, Dedan Githae, Spyridon Gkotzis, Liliana Godoy, Samantha Goldman, Gaston H. Gonnet, Juana Gonzalez, Andrea Gonzalez, Camila Gonzalez-Poblete, Andrew Gray, Tranette Gregory, Charlotte Greselle, Sophie Guasco, Juan Guerra, Nika Gurianova, Wolfgang Haehr, Sebastien Halary, Felix Hartkopf, Jaden J.A. Hastings, Arya Hawkins-Zafarnia, Nur Hazlin Hazrin-Chong, Eric Helfrich, Eva Hell, Tamera Henry, Samuel Hernandez, Pilar Lopez Hernandez, David Hess-Homeier, Lauren E. Hittle, Nghiem Xuan Hoan, Aliaksei Holik, Chiaki Homma, Irene Hoxie, Michael Huber, Elizabeth Humphries, Stephanie Hyland, Andrea Hässig, Roland Häusler, Nathalie Hüsser, Robert A. Petit, Badamnyambuu Iderzorig, Mizuki Igarashi, Shaikh B. Iqbal, Shino Ishikawa, Sakura Ishizuka, Sharah Islam, Riham Islam, Kohei Ito, Sota Ito, Takayuki Ito, Tomislav Ivankovic, Tomoki Iwashiro, Sarah Jackson, JoAnn Jacobs, Marisano James, Marianne Jaubert, Marie-Laure Jerier, Esmeralda Jiminez, Ayantu Jinfessa, Ymke De Jong, Hyun Woo Joo, Guilllaume Jospin, Takema Kajita, Affifah Saadah Ahmad Kassim, Nao Kato, Amrit Kaur, Inderjit Kaur, Fernanda de Souza Gomes Kehdy, Vedbar S. Khadka, Shaira Khan, Mahshid Khavari, Michelle Ki, Gina Kim, Hyung Jun Kim, Sangwan Kim, Ryan J. King, Kaymisha Knights, Giuseppe KoLoMonaco, Ellen Koag, Nadezhda Kobko-Litskevitch, Maryna Korshevniuk, Michael Kozhar, Jonas Krebs, Nanami Kubota, Andrii Kuklin, Sheelta S. Kumar, Rachel Kwong, Lawrence Kwong, Ingrid Lafontaine, Juliana Lago, Tsoi Ying Lai, Elodie Laine, Manolo Laiola, Olha Lakhneko, Isha Lamba, Gerardo de Lamotte, Romain Lannes, Eleonora De Lazzari, Madeline Leahy, Hyunjung Lee, Yunmi Lee, Lucy Lee, Vincent Lemaire, Emily Leong, Marcus H.Y. Leung, Dagmara Lewandowska, Chenhao Li, Weijun Liang, Moses Lin, Priscilla Lisboa, Anna Litskevitch, Eric Minwei Liu, Tracy Liu, Mayra Arauco Livia, Yui Him Lo, Sonia Losim, Manon Loubens, Jennifer Lu, Olexandr Lykhenko, Simona Lysakova, Salah Mahmoud, Sara Abdul Majid, Natalka Makogon, Denisse Maldonado, Krizzy Mallari, Tathiane M. Malta, Maliha Mamun, Dimitri Manoir, German Marchandon, Natalia Marciniak, Sonia Marinovic, Brunna Marques, Nicole Mathews, Yuri Matsuzaki, Vincent Matthys, Madelyn May, Elias McComb, Annabelle Meagher, Adiell Melamed, Wayne Menary, Katterinne N. Mendez, Ambar Mendez, Irène Mauricette Mendy, Irene Meng, Ajay Menon, Mark Menor, Roy Meoded, Nancy Merino, Cem Meydan, Karishma Miah, Mathilde Mignotte, Tanja Miketic, Wilson Miranda, Athena Mitsios, Ryusei Miura, Kunihiko Miyake, Maria D. Moccia, Natasha Mohan, Mohammed Mohsin, Karobi Moitra, Mauricio Moldes, Laura Molina, Jennifer Molinet, Orgil-Erdene Molomjamts, Eftar Moniruzzaman, Sookwon Moon, Isabelle de Oliveira Moraes, Mario Moreno, Maritza S. Mosella, Josef W. Moser, Christopher Mozsary, Amanda L. Muehlbauer, Oasima Muner, Muntaha Munia, Naimah Munim, Maureen Muscat, Tatjana Mustac, Cristina Muñoz, Francesca Nadalin, Areeg Naeem, Dorottya Nagy-Szakal, Mayuko Nakagawa, Ashanti Narce, Masaki Nasu, Irene González Navarrete, Hiba Naveed, Bryan Nazario, Narasimha Rao Nedunuri, Thomas Neff, Aida Nesimi, Wan Chiew Ng, Synti Ng, Gloria Nguyen, Elsy Ngwa, Agier Nicolas, Pierre Nicolas, Abdollahi Nika, Hosna Noorzi, Avigdor Nosrati, Houtan Noushmehr, Diana N. Nunes, Kathryn O’Brien, Niamh B. O’Hara, Gabriella Oken, Rantimi A. Olawoyin, Javier Quilez Oliete, Kiara Olmeda, Tolulope Oluwadare, Itunu A. Oluwadare, Nils Ordioni, Jenessa Orpilla, Jacqueline Orrego, Melissa Ortega, Princess Osma, Israel O. Osuolale, Oluwatosin M. Osuolale, Mitsuki Ota, Francesco Oteri, Yuya Oto, Rachid Ounit, Christos A. Ouzounis, Subhamitra Pakrashi, Rachel Paras, Coral Pardo-Este, Young-Ja Park, Paulina Pastuszek, Suraj Patel, Jananan Pathmanathan, Andrea Patrignani, Manuel Perez, Ante Peros, Sabrina Persaud, Anisia Peters, Adam Phillips, Lisbeth Pineda, Melissa P. Pizzi, Alma Plaku, Alketa Plaku, Brianna Pompa-Hogan, María Gabriela Portilla, Leonardo Posada, Max Priestman, Bharath Prithiviraj, Sambhawa Priya, Phanthira Pugdeethosal, Catherine E. Pugh, Benjamin Pulatov, Angelika Pupiec, Kyrylo Pyrshev, Tao Qing, Saher Rahiel, Savlatjon Rahmatulloev, Kannan Rajendran, Aneisa Ramcharan, Adan Ramirez-Rojas, Shahryar Rana, Prashanthi Ratnanandan, Timothy D. Read, Hubert Rehrauer, Renee Richer, Alexis Rivera, Michelle Rivera, Alessandro Robertiello, Courtney Robinson, Paula Rodríguez, Nayra Aguilar Rojas, Paul Roldán, Anyelic Rosario, Sandra Roth, Maria Ruiz, Stephen Eduard Boja Ruiz, Kaitlan Russell, Mariia Rybak, Thais S. Sabedot, Mahfuza Sabina, Ikuto Saito, Yoshitaka Saito, Gustavo Adolfo Malca Salas, Cecilia Salazar, Kaung Myat San, Jorge Sanchez, Khaliun Sanchir, Ryan Sankar, Paulo Thiago de Souza Santos, Zulena Saravi, Kai Sasaki, Yuma Sato, Masaki Sato, Seisuke Sato, Ryo Sato, Kaisei Sato, Nowshin Sayara, Steffen Schaaf, Oli Schacher, Anna-Lena M. Schinke, Ralph Schlapbach, Christian Schori, Jason R. Schriml, Felipe Segato, Felipe Sepulveda, Marianna S. Serpa, Paola F. De Sessions, Juan C. Severyn, Heba Shaaban, Maheen Shakil, Sarah Shalaby, Aliyah Shari, Hyenah Shim, Hikaru Shirahata, Yuh Shiwa, Rania Siam, Ophélie Da Silva, Jordana M. Silva, Gwenola Simon, Shaleni K. Singh, Kasia Sluzek, Rebecca Smith, Eunice So, Núria Andreu Somavilla, Yuya Sonohara, Nuno Rufino de Sousa, Camila Souza, Jason Sperry, Nicolas Sprinsky, Stefan G. Stark, Antonietta La Storia, Kiyoshi Suganuma, Hamood Suliman, Jill Sullivan, Arif Asyraf Md Supie, Chisato Suzuki, Sora Takagi, Fumie Takahara, Naoya Takahashi, Kou Takahashi, Tomoki Takeda, Isabella K. Takenaka, Soma Tanaka, Anyi Tang, Yuk Man Tang, Emilio Tarcitano, Andrea Tassinari, Mahdi Taye, Alexis Terrero, Eunice Thambiraja, Antonin Thiébaut, Sade Thomas, Andrew M. Thomas, Yuto Togashi, Takumi Togashi, Anna Tomaselli, Masaru Tomita, Itsuki Tomita, Xinzhao Tong, Oliver Toth, Nora C. Toussaint, Jennifer M. Tran, Catalina Truong, Stefan I. Tsonev, Kazutoshi Tsuda, Takafumi Tsurumaki, Michelle Tuz, Yelyzaveta Tymoshenko, Carmen Urgiles, Mariko Usui, Sophie Vacant, Brandon Valentine, Laura E. Vann, Fabienne Velter, Valeria Ventorino, Patricia Vera-Wolf, Riccardo Vicedomini, Michael A. Suarez-Villamil, Sierra Vincent, Renee Vivancos-Koopman, Andrew Wan, Cindy Wang, Tomoro Warashina, Ayuki Watanabe, Samuel Weekes, Johannes Werner, David Westfall, Lothar H. Wieler, Michelle Williams, Silver A. Wolf, Brian Wong, Yan Ling Wong, Tyler Wong, Rasheena Wright, Tina Wunderlin, Ryota Yamanaka, Jingcheng Yang, Hirokazu Yano, George C. Yeh, Olena Yemets, Tetiana Yeskova, Shusei Yoshikawa, Laraib Zafar, Yang Zhang, Shu Zhang, Amy Zhang, Yuanting Zheng, Stas Zubenko

**Affiliations:** 1 Weill Cornell Medicine, New York, NY, USA; 2 The Bin Talal Bin Abdulaziz Alsaud Institute for Computational Biomedicine, New York, NY, USA; 3 Icahn School of Medicine at Mount Sinai, New York, NY, USA; 4 Genome Institute of Singapore, A^∗^STAR, Singapore, Singapore; 5 Centre for Genomic Regulation (CRG), The Barcelona Institute of Science and Technology, Barcelona, Spain; 6 ETH Zurich, Department of Computer Science, Biomedical Informatics Group, Zurich, Switzerland; 7 Department of Energy, Joint Genome Institute, Lawrence Berkeley National Laboratory, Berkeley, CA, USA; 8 Kenya Medical Research Institute – Kisumu, Kisumu, Kenya; 9 Independent Researcher, Zurich, Switzerland; 10 Massachusetts Institute of Technology, McGovern Institute for Brain Research, Cambridge, MA, USA; 11 Machine Intelligence Unit, Indian Statistical Institute, Kolkata, India; 12 Centre for Artificial Intelligence and Machine Learning, Indian Statistical Institute, Kolkata, India; 13 University of Minnesota, Minneapolis, MN, USA; 14 Universidad Andres Bello, Center for Bioinformatics and Integrative Biology, Facultad de Ciencias de la Vida, Santiago, Chile; 15 California State University, Sacramento, Sacramento, CA, USA; 16 Department of Agricultural Sciences, Division of Microbiology, University of Naples Federico II, Naples, Italy; 17 Task Force on Microbiome Studies, University of Naples Federico II, Naples, Italy; 18 University of Hawaii John A. Burns School of Medicine, Honolulu, HI, USA; 19 Aix-Marseille Université, Mediterranean Institute of Oceanology, Université de Toulon, CNRS, IRD, UM 110, Marseille, France; 20 Medical Genomics group, A.C.Camargo Cancer Center, São Paulo – SP, Brazil; 21 Norwegian Defence Research Establishment FFI, Kjeller, Norway; 22 Department of Biology, Lund University, Lund, Sweden; 23 Institute of Molecular Biology and Genetics of National Academy of Sciences of Ukraine, Kyiv, Ukraine; 24 University of Applied Sciences Vienna, Vienna, Austria; 25 Department of Analytical, Environmental and Forensic Sciences, King's College London, London, UK; 26 University of Colorado at Boulder, Boulder, CO, USA; 27 Microbial Genomics Laboratory, Institut Pasteur de Montevideo, Montevideo, Uruguay; 28 Center for Integrative Biology, Universidad Mayor, Santiago de Chile, Santiago, Chile; 29 Wellcome Sanger Institute, Hinxton, UK; 30 Institut Pasteur Korea, Seoul, South Korea; 31 School of Energy and Environment, City University of Hong Kong, Hong Kong SAR, China; 32 Department of Molecular Biosciences, The Wenner-Gren Institute, Stockholm University, Stockholm, Sweden; 33 Microba, 388 Queen St, Brisbane City, QLD 4000, Australia; 34 University of Maryland School of Medicine, Institute for Genome Sciences, Baltimore, MD, USA; 35 Fundação Oswaldo Cruz, Rio de Janeiro – RJ, Brazil; 36 University of São Paulo, Ribeirão Preto Medical School, Ribeirão Preto – SP, Brazil; 37 Instituto de Patologia e Imunologia Molecular da Universidade do Porto, Porto, Portugal; 38 Institute of Medical Genetics and Applied Genomics, University of Tübingen, Tübingen, Germany; 39 NGS Competence Center Tübingen (NCCT), University of Tübingen, Tübingen, Germany; 40 Applied Environmental Metagenomics and Infectious Diseases Research (AEMIDR), Department of Biological Sciences, Elizade University, Ilara-Mokin, Nigeria; 41 Microbial Paleogenomics Unit, Institut Pasteur, CNRS UMR2000, Paris 75015, France; 42 Sorbonne University, Faculty of Science, Institute of Biology Paris-Seine, Laboratory of Computational and Quantitative Biology, Paris, France; 43 Robert Koch Institute, Berlin, Germany; 44 Acibadem Mehmet Ali Aydınlar University, Istanbul, Turkey; 45 Center for Pharmacogenomics, School of Life Sciences and Shanghai Cancer Center, Fudan University, Shanghai, China; 46 State Key Laboratory of Genetic Engineering (SKLGE) and MOE Key Laboratory of Contemporary Anthropology, School of Life Sciences, Human Phenome Institute, Fudan University, Shanghai, China; 47 The Center for Bioinformatics and Computational Biology, Shanghai Key Laboratory of Regulatory Biology, the Institute of Biomedical Sciences and School of Life Sciences, East China Normal University, Shanghai, China; 48 108 Military Central Hospital, Hanoi, Vietnam; 49 Vietnamese-German Center for Medical Research (VG-CARE), Hanoi, Vietnam; 50 Keio University, Tokyo, Japan; 51 University of Vermont, Burlington, VT, USA; 52 Millennium Initiative for Collaborative Research on Bacterial Resistance, Santiago, Chile; 53 Faculty of Mathematics and Informatics, Sofia University “St. Kliment Ohridski,” Sofia, Bulgaria; 54 Institute of Arctic Biology, University of Alaska, Fairbanks, Fairbanks, AK, USA; 55 Institute of Tropical Medicine, Univeristätsklinikum Tübingen, Tübingen, Germany; 56 Faculty of Medicine, Duy Tan University, Da Nang, Vietnam; 57 Corporación Corpogen-Research Center, Bogotá, Colombia; 58 Department of Epidemiology, School of Public Health, Fudan University, Shanghai, China; 59 Małopolska Centre of Biotechnology, Jagiellonian University, Kraków, Poland; 60 Boku University Viennna, Vienna, Austria; 61 SciLife EVP, Department of Aquatic Sciences Assessment, Swedish University of Agricultural Sciences, Uppsala, Sweden; 62 Kyiv Academic University, Kyiv, Ukraine; 63 C+, Research Center in Technologies for Society, School of Engineering, Universidad del Desarrollo, Santiago, Chile; 64 University Hospital Zurich, Biomedical Informatics Research, Zurich, Switzerland; 65 Swiss Institute of Bioinformatics, Lausanne, Switzerland; 66 University of Medicine and Health Sciences, St. Kitts, West Indies and American University in Cairo, Cairo, Egypt; 67 The WorldQuant Initiative for Quantitative Prediction, Weill Cornell Medicine, New York, NY, USA

**Keywords:** built Environment, metagenome, global health, antimicrobial resistance, microbiome, NGS, shotgun sequencing, de novo assembly, BGC, AMR

## Abstract

We present a global atlas of 4,728 metagenomic samples from mass-transit systems in 60 cities over 3 years, representing the first systematic, worldwide catalog of the urban microbial ecosystem. This atlas provides an annotated, geospatial profile of microbial strains, functional characteristics, antimicrobial resistance (AMR) markers, and genetic elements, including 10,928 viruses, 1,302 bacteria, 2 archaea, and 838,532 CRISPR arrays not found in reference databases. We identified 4,246 known species of urban microorganisms and a consistent set of 31 species found in 97% of samples that were distinct from human commensal organisms. Profiles of AMR genes varied widely in type and density across cities. Cities showed distinct microbial taxonomic signatures that were driven by climate and geographic differences. These results constitute a high-resolution global metagenomic atlas that enables discovery of organisms and genes, highlights potential public health and forensic applications, and provides a culture-independent view of AMR burden in cities.

## Highlights

• Cities possess a consistent “core” set of non-human microbes
• Urban microbiomes echo important features of cities and city-life
• Antimicrobial resistance genes are widespread in cities
• Cities contain many novel bacterial and viral species

## Graphical abstract

**Figure:**
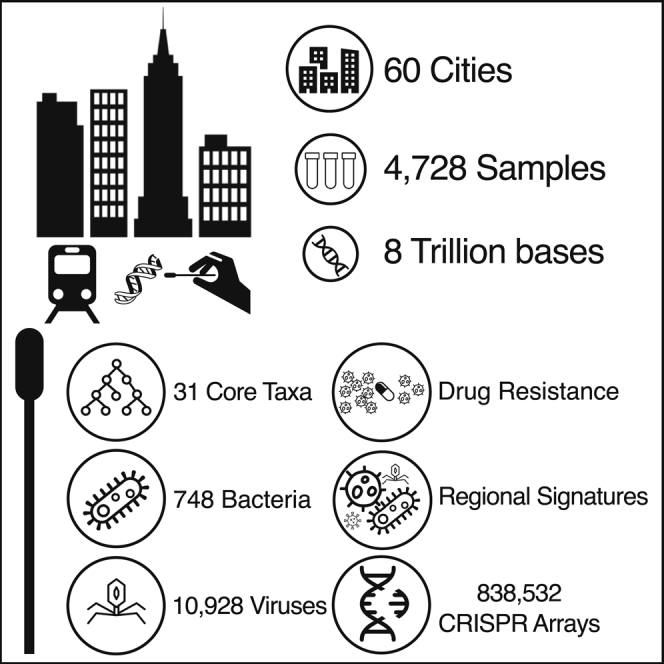

## Teaser

This systematic, worldwide catalog of urban microbiomes represents a metagenomic atlas important for understanding the ecology, virulence, and antibiotic resistance of city-specific microbial communities.

## Introduction

The high-density urban environment has historically been home to only a fraction of all people, with the majority living in rural areas or small villages. In the last two decades, the situation has reversed; 55% of the world’s population now lives in urban areas (Ritchie and Roser, 2020; United Nations, 2018). Since the introduction of germ theory and John Snow’s work on cholera, it has been clear that people in cities interact with microbes in ways that can be markedly different than in rural areas (Neiderud, 2015). Microbes in the built environment have been implicated as a possible source of contagion (Cooley et al., 1998) and certain syndromes, such as allergies, are associated with increasing urbanization (Nicolaou et al., 2005). It is now apparent that cities, in general, have an impact on human health, though the mechanisms of this impact are broadly variable and often little understood. Indeed, our understanding of microbial dynamics in the urban environment outside of pandemics has only just begun (Gilbert and Stephens, 2018).

Technological advances in next-generation sequencing (NGS) and metagenomics have created an unprecedented opportunity for rapid, global studies of microorganisms and their hosts, providing researchers, clinicians, and policymakers with a more comprehensive view of the functional dynamics of microorganisms in a city. NGS facilitates culture-independent sampling of the microorganisms in an area with the potential for both taxonomic and functional annotation; this is particularly important for surveillance of microorganisms as they acquire antimicrobial resistance (AMR) (Afshinnekoo et al., 2021; Fresia et al., 2019). Metagenomic methods enable nearly real-time monitoring of organisms, AMR genes, and pathogens as they emerge within a given geographical location and have the potential to reveal hidden microbial reservoirs and detect microbial transmission routes as they spread around the world (Zhu et al., 2017). There are several different drivers and sources for AMR, including agriculture, farming, and livestock in rural and suburban areas; household and industrial sewage; usage of antimicrobials, hard metals, and biocides; as well as human and animal waste. All these factors contribute to the complexity of AMR transmission (Allen et al., 2009; Martínez, 2008; Singer et al., 2016; Thanner et al., 2016; Venter et al., 2017). A molecular map of urban environments will enable significant new research on the impact of urban microbiomes on human health.

Urban transit systems—including subways and buses—are a daily contact interface for billions of people who live in cities. Urban travelers bring their commensal microorganisms with them as they travel and come into contact with organisms and mobile elements present in the environment. The study of the urban microbiome and the microbiome of the built environment spans several different projects and initiatives, including work focused on transit systems (Afshinnekoo et al., 2015; Hsu et al., 2016; Kang et al., 2018; Leung et al., 2014; MetaSUB International Consortium et al., 2016), hospitals (Brooks et al., 2017; Lax et al., 2017), soil (Hoch et al., 2019; Joyner et al., 2019), and sewage (Fresia et al., 2019; Maritz et al., 2019), among others. For the most part, these efforts have only studied a few select cities on a limited number of occasions. This leaves a gap in scientific knowledge about a microbial ecosystem with which the global human population readily interacts. Human commensal microbiomes have also been found to vary based on culture, and thus geographically isolated studies are limited and may miss key differences (Brito et al., 2016). Moreover, data on urban microbes and AMR genes are urgently needed in developing nations, where antimicrobial drug consumption is expected to rise by 67% by 2030 (United Nations, 2016; Van Boeckel et al., 2015), both from changes in consumer demand for livestock products and expanding use of antimicrobials—both of which can alter AMR profiles of these cities.

The International Metagenomics and Metadesign of Subways and Urban Biomes (MetaSUB) Consortium was launched in 2015 to address this gap in knowledge on the density, types, and dynamics of urban metagenomes and AMR profiles. Since then, we have developed standardized collection and sequencing protocols to process 4,728 samples across 60 cities worldwide (Table S1). Sampling took place at three major time points: a pilot study in 2015–2016 and two global city sampling days (June 21st) in 2016 and 2017. Each sample was sequenced with 5–7 million 125bp paired-end reads using Illumina NGS sequencers (see STAR Methods). To deal with the challenging analysis of our large dataset, we generated an open-source analysis pipeline (MetaSUB Core Analysis Pipeline, CAP), which includes a comprehensive set of state-of-the-art, peer-reviewed, metagenomic tools for taxonomic identification, k-mer analysis, AMR gene prediction, functional profiling, *de novo* assembly, taxon annotation, and geospatial mapping. To our knowledge, this study represents the first extensive global metagenomic study of urban microbiomes. This study reveals a consistent “core” urban microbiome across all cities, as well as distinct geographic variation that may reflect the epidemiological variation and that enables a new forensic, city-specific source-tracking. Our data demonstrate a significant fraction of the urban microbiome remains to be characterized. Though 1,000 samples are sufficient to discover roughly 80% of the observed taxa and AMR markers, we continued to observe taxa and genes not found in other samples. This genetic variation is affected by environmental factors (e.g., climate, surface type, latitude, etc.), and samples show greater diversity near the equator. Sequences associated with AMR markers are widespread, though not necessarily abundant, and show geographic specificity. Here, we present the results of our global analyses and a set of tools developed to access and analyze this extensive atlas, including two interactive map-based visualizations for samples (metasub.org/map) and AMRs (resistanceopen.org), an indexed search tool over raw sequence data (https://metagraph.ethz.ch/search), a Git repository for all analytical pipelines and figures, and application programming interfaces (APIs) for computationally accessing results (https://github.com/metasub/metasub_utils).

## Results

We collected 4,728 samples from the mass transit systems of 60 cities around the world (Table 1; Table S1). These samples were collected from at least three common surfaces in each mass transit system (railings, benches, and ticket kiosks), with additional optional surfaces also collected in each city, and all were subjected to shotgun metagenomic sequencing (125 × 125 PE reads, see STAR Methods). We use the microbiome of mass transit systems as a proxy for the urban microbiome as a whole and present our key findings here.

**Table 1:** Sample counts

Region	Pilot	CSD16	CSD17	Other	Total
North America	28	284	371	276	959
East Asia	34	26	1,297	0	1,357
Europe	177	310	939	1	1,427
Sub-Saharan Africa	0	116	192	0	308
South America	20	44	199	68	331
Middle East	0	100	15	0	115
Oceania	0	94	32	0	126
Background control	0	0	40	0	40
Lab control	0	0	20	6	26
Positive control	0	0	33	6	39
Total	259	974	3,138	357	4,728

The number of samples collected from each region.

### A core urban microbiome centers global diversity

**Figure 1:**
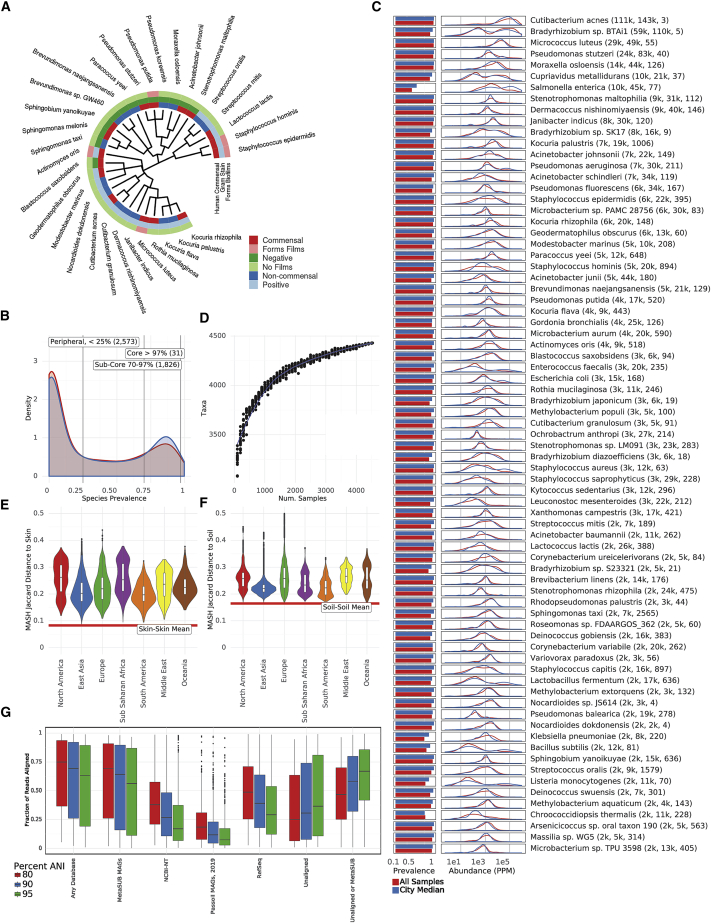
The core microbiome (A) Taxonomic tree showing 31 core taxa, annotated according to gram stain, ability to form biofilms, and whether the bacteria is a human commensal species. (B) Distribution of species prevalence from all samples and normalized by cities. Vertical lines show defined group cutoffs. (C) Prevalence and distribution of relative abundances of the 75 most abundant taxa. Mean relative abundance, standard deviation, and kurtosis of the abundance distribution are shown. (D) Rarefaction analysis showing the number of species detected in randomly chosen sets of samples. (E) MASH (k-mer-based) similarity between MetaSUB samples and HMP skin microbiome samples by continent. (F) MASH (k-mer based) similarity between MetaSUB samples and soil microbiome samples by continent. (G) Fraction of reads aligned (via BLAST) to different databases at different average nucleotide identities. See also Figure S1.

We first investigated the distribution of microbial species across the global urban environment. Specifically, we asked whether the urban environment represents a singular type of microbial ecosystem or a set of related but distinct communities, especially in terms of biodiversity. We observed a bimodal distribution of taxa prevalence across our dataset, which we used to define two separate sets of taxa based on the inflection points of the distribution: the putative “sub-core” set of urban microbial species that are consistently observed (>70% of samples) and the less common “peripheral” (<25% of samples) species. We also defined a set of true “core” taxa, which occur in essentially all samples (>97% of samples) (Figure 1A). Applying these thresholds, we identified 1,145 microbial species (Figure 1B), as defined by the NCBI annotation in KrakenUniq, that make up the sub-core urban microbiome with 31 species in the true core microbiome (Figure 1A). Core and sub-core taxa classifications were further evaluated for sequence complexity and genome coverage on a subset of samples. Of the sub-core species, 69 were flagged as being low-quality classifications (see STAR Methods). The sub-core microbiome was principally bacterial, with just one high-confidence eukaryote identified: *Saccharomyces cerevisiae*. Notably, no archaea or viruses were identified in the group of sub-core microorganisms. For viruses in particular, this may be affected by the DNA extraction methods used, limitations in sequencing depth, or missing annotations in reference databases used for classification. The three most common bacterial phyla across the world’s cities ordered by the number of species observed were *Proteobacteria*, *Actinobacteria*, and *Firmicutes*.

Despite their global prevalence, the core taxa were not uniformly abundant across all cities. Many species exhibited a high standard deviation and kurtosis (Fisher’s definition) relative to other species (Figure 1C). Some species showed distinctly high mean abundance, often higher than core species, but more heterogeneous global prevalence. For example, *Salmonella enterica* was identified in *<*50% of samples but was the 12th most abundant species based on the fraction of DNA ascribed to it. The most relatively abundant microbial species was *Cutibacterium acnes* (Figure 1D), which had a comparatively stable distribution of abundance across all samples, and is a known human skin commensal. To correct for bias arising from uneven geographic sampling, we measured the relative abundance of each taxon by calculating the fraction of reads classified to each taxon and compared the raw distribution to the distribution of median abundances within each city; the two measures closely aligned. An examination of the positive and negative controls indicates that these results are not likely due to contamination or batch effect (see STAR Methods). In total, we observed 31 core taxa (>97% prevalence), 1,145 sub-core taxa (70%–97% prevalence), 2,466 peripheral taxa (<25% prevalence), and 4,424 taxa across all samples. We term the set of all high-confidence taxa observed in *the urban panmicrobiome*.

To estimate the number of taxa present in our samples but that may have been missed by our methods (e.g., sampling type and sequencing depth), we performed a rarefaction analysis on the taxa that were identified. By estimating the number of taxa identified for different numbers of samples as a function of the number of reads, we see a diminishing trend (Figure 1D), which indicates that at some point, the species in every new sample were likely already identified in a previous one. Our rarefaction curve did not reach a plateau, and even after including all samples, it still showed a marginal discovery rate of roughly one new species for every 10 samples added to the study. For clarity, we note that this analysis only considers taxa already present in reference databases, not newly discovered taxa (below). Despite the remaining unidentified taxa, we estimate that most (80%) of the classifiable taxa in the urban microbiome could be identified with roughly 1,000 samples.

**Figure S1:**
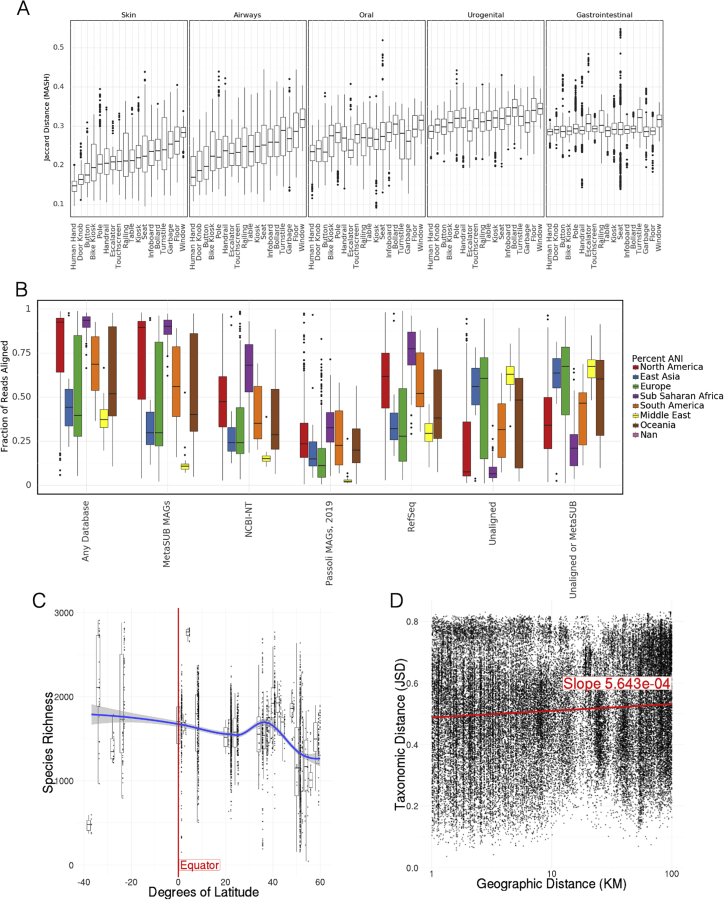
Core urban taxa and ecological trends, related to Figure 1 (A) Jaccard similarity of MASH indices to HMP samples for different surface types. (B) Fraction of reads assigned at 80% ANI to different databases by BLAST for each region. (C) Correlation between species richness and latitude. Richness decreases significantly with latitude. (D) Neighborhood effect. Taxonomic distance weakly correlates with geographic distance within cities.

Since humans are a major part of the urban environment, the DNA in our samples could be expected to resemble commensal human microbiomes. To investigate this, we compared non-human DNA fragments from our samples to 50 randomly selected samples from five commensal microbiome sites (stool, skin, airway, gastrointestinal tract, urogenital tract; 10 samples of each type) in the Human Microbiome Project (HMP) (Consortium et al., 2012). We used MASH to perform a k-mer-based comparison of our samples versus the selected HMP samples, which showed a roughly uniform dissimilarity between MetaSUB samples and those from different human body sites (Figures 1E and S1A). Samples taken from surfaces that were likely to have been touched more often by human skin, such as doorknobs, buttons, railings, and touchscreens, were indeed more similar to the human skin microbiomes than surfaces like bollards, windows, and the floor. For example, doorknobs were significantly more similar to skin than windows (t test, p < 2e-16).

We performed an analogous comparison to a set of 28 metagenomic soil samples (Bahram et al., 2018). Our samples were more dissimilar from the soil samples (Figure 1F) than they were to human skin microbiomes. This suggests that unclassified DNA in our samples may represent uncharacterized taxa that are not known commensal or soil species.

We next estimated the fraction of sequences in our data that did not resemble sequences in known reference databases. We took a subset of 10,000 reads from each sample and aligned these reads to four large-scale sequence databases using BLASTn (Altschul et al., 1990). We identified reads that mapped at 80%, 90%, and 95% average nucleotide identity (ANI) (Figure 1G) to sequences in the RefSeq reference database, NCBI’s NT Environmental database, a large set of Metagenome Assembled Genomes (MAGs) from Pasolli et al. (2019), and MAGs from MetaSUB itself (see widespread observation of biology not in reference databases). At 80% ANI, the most permissive threshold, we observed that 34.6% of reads did not map to any database, while 47.3% of reads did not map to any database except MAGs from MetaSUB itself. This mirrors results seen by previous urban microbiome works (Afshinnekoo et al., 2015; Hsu et al., 2016). When we broke alignment rates down by region, we found that samples from Europe had the highest fraction of unaligned reads, followed by the Middle East, while samples from Sub-Saharan Africa had the smallest fraction of unaligned reads (Figure S1B).

Previous ecological studies have observed a decrease in taxonomic diversity as the distance from the equator increases (O’Hara et al., 2017). Our data recapitulated this result and identify a significant decrease in taxonomic diversity (though with significant noise, p < 2*e*16, *R*^2^ = 0.06915) as a function of absolute latitude; samples are estimated to lose 6.97 species for each degree of latitude away from the equator (Figure S1C). While this is an observation consistent with ecological theory, we note that our samples are somewhat clustered in specific latitudes.

### Global diversity varies according to key covariates

**Figure 2:**
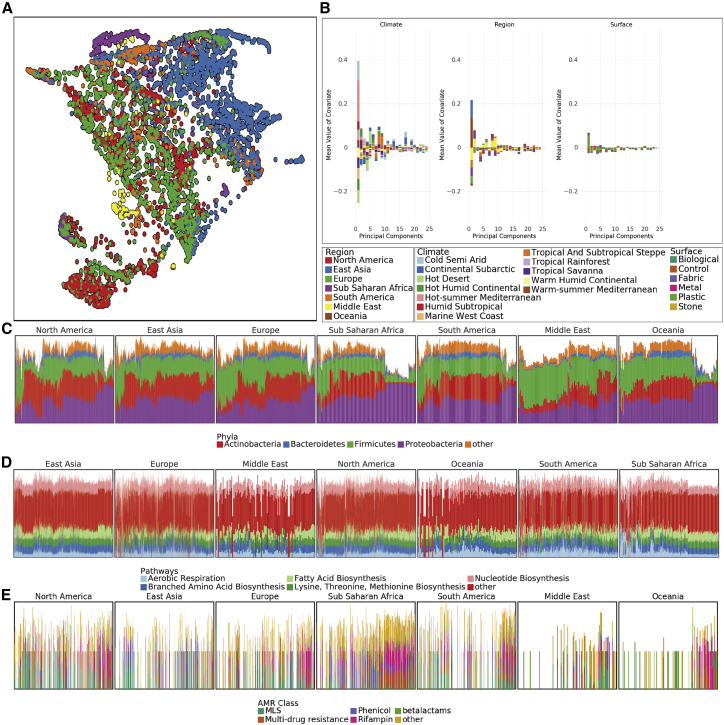
Differences at global scale (A) UMAP of taxonomic profiles based on Jaccard distance between samples. Colored by the region of origin for each sample. Axes are arbitrary and without meaningful scale. The color key is shared with (B). (B) Association of the first 25 principal components of sample taxonomy with climate, continent, and surface material. (C) Distribution of ma1jo0r phyla, sorted by hierarchical clustering of all samples and grouped by continent. (D) Distribution of high-level groups of functional pathways, using the same order as taxa (C). (E) Distribution of AMR genes by drug class (as defined in MegaRes), using the same order as taxa (C). Note that MLS is macrolide-lincosamide-streptogramin. See also Figure S3.

**Figure S2:**
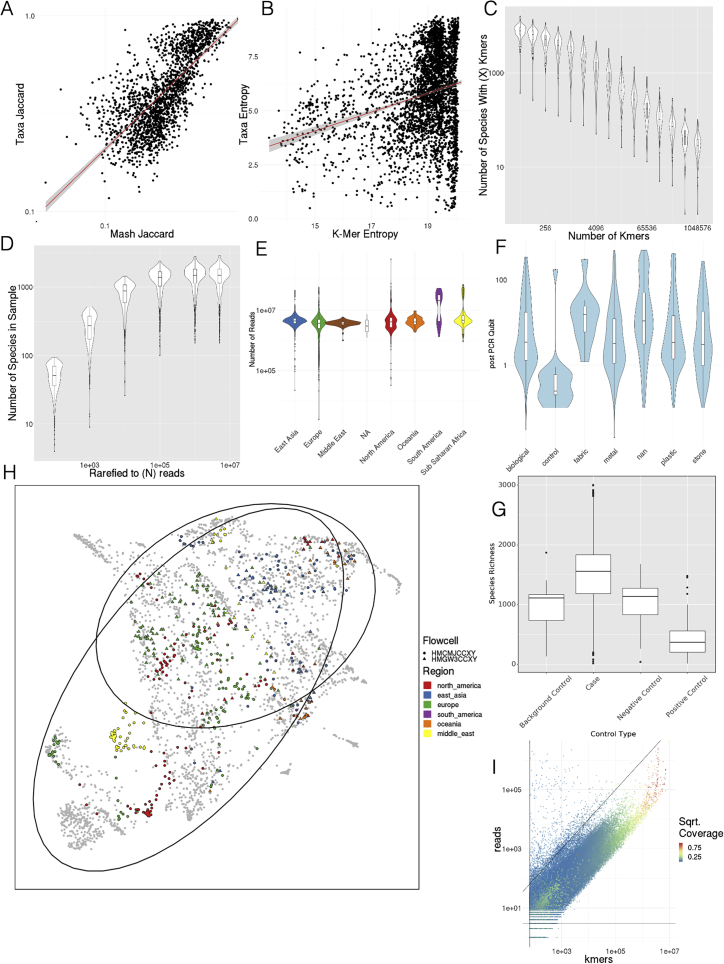
Quality control and metrics, related to Figures 1 and 2 (A) Jaccard distance of taxonomic profiles versus MASH Jaccard distance of k-mers. (B) Shannon’s Entropy of taxonomic profiles versus Shannon’s Entropy of k-mers. (C) Number of species detected as k-mer threshold increases for 100 randomly selected samples. (D) Number of species detected as number of sub-sampled reads increase. (E) Number of reads by region. (F) PCR Qubit by surface material. (G) Taxonomic Richness in Cases versus Types of Controls. (H) Flowcells versus quality control metrics See also Methods. (I) k-mer counts compared to number of reads for species level annotations in 100 randomly selected samples, colored by coverage of marker k-mer set.

Despite the core urban microbiome present in almost all samples, there was nonetheless a wide range of variation in taxonomy and localization across all the cities. To quantify this, we calculated the Jaccard distance between samples based on the presence and absence of all panmicrobiome species and performed a dimensionality reduction of the data using UMAP (uniform manifold approximation and projection, McInnes et al., 2018) for visualization (Figure 2A). In principle, Jaccard distance could be influenced by read depth, where low abundance species drop below the detection threshold. However, we expect this issue to be minor as Jaccard distance of taxonomic profiles correlated with k-mer-based distances (Figures S2A and S2B) and because the total number of species identified stabilized at roughly 100,000 reads (with no sharp quality drop-off; Figures S2C and S2D) compared to an average of 6.01 million reads per sample.

**Figure S3:**
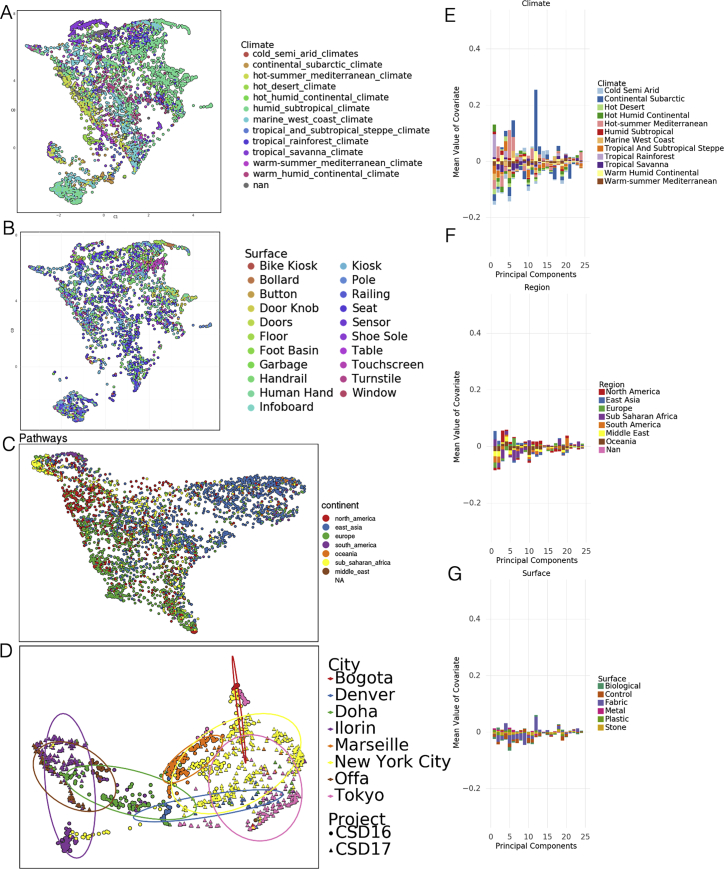
Diversity and variation, related to Figure 2 (A) UMAP of taxonomic profiles colored by climate classification. (B) UMAP of taxonomic profiles colored by surface type. (C) UMAP of functional profiles colored by region. (D) Taxonomic shift over time in cities with two years of sampling. UMAP dimensionality reduction of taxonomic profiles for each sample shows variation within cities across time (2016, circles and 2017, triangles) though generally less variation than between cities (colors). (E–G) Sources of variation for AMRs. Association of the first 25 principal components of AMR genes with climate, region, and surface material.

Since taxonomic profiles from North America and Europe were distinct from those collected in East Asia (with smaller clusters for other regions), we next examined variation as function of functional classification, climate, surface type, and year of sampling. Subclusters identified by UMAP of taxonomic profiles roughly corresponded to climate but not surface type (Figures S3A and S3B). Similar to taxonomy, dimensionality reduction of functional metabolic profiles showed a geospatial difference between regions (Figure S3C), indicating stratification of the metagenomes at both the functional and genus/species levels. These findings confirm and extend earlier analyses performed on a fraction of the MetaSUB data, which were run as a part of CAMDA Challenges (camda.info). To gauge the impact of time, we also compared variation in matched sites from cities with two consecutive years of sampling on the summer solstice (June 21). While taxonomic change within a city between 2016 and 2017 was usually less than the difference between cities (Figure S3D), this may become a more important factor over longer time periods.

We next quantified the degree to which metadata covariates influence the taxonomic composition of our samples using MAVRIC, a statistical tool to estimate the sources of variation in a count-based dataset (Moskowitz and Greenleaf, 2018) according to each samples’ metadata of city, population density, average temperature in June, region, elevation above sea level, surface type, surface material, elevation above or below ground, and proximity to the coast. The most important factor (19% of the variation) was the city from which a sample was taken, followed by the world’s overall region (11%). The other four factors accounted for 2% to 7% of the possible variation in taxonomy (Table S2). We note that many of the factors were confounded with one another, so they can explain less diversity than their sum. Of note, the population density of the sampled city had no significant effect on taxonomic variation.

Given this strong signal from each city, we performed a principal component analysis (PCA) on our taxonomic data, normalized by the proportion of identified principal components (PCs) that were associated with a metadata covariate (positive or negative). We hypothesized that some principle covariates, such as climate, continent, and surface material, might be prominent factors driving the taxonomic composition of a given sample. We found that the two most prominent absolute PCs associated strongly with the city climate (representing 28.0% and 15.7% of the variance of the original data, respectively), while the continent and surface material associated less strongly (Figure 2B); the same trend held for the variation of AMR genes (Figures S3E–S3G) as well.

We tested if samples that were close together in cities were more similar to one another. For pairs of samples taken in the same city, the geographic distance between samples was crudely predictive of the Jensen-Shannon distance between taxonomic profiles. Every increase of 1 km in distance between two samples represented an increase of 0.056% in divergence (p < 2*e*16, *R*^2^ = 0.01073; Figure S1D). To reduce potential bias from samples taken from the same object, we excluded all pairs of samples within 1 km of one another. This suggests a “neighborhood effect” for sample similarity analogous to the effect described by Meyer et al. (2018), albeit a very minor one.

At a global level, we examined the prevalence and abundance of taxa and their functional profiles between cities and continents. These data showed that most samples contained species from four phyla: *Actinobacteria*, *Bacteroidetes*, *Firmicutes*, and *Proteobacteria*, but that the relative abundance of these phyla varied (Figure 2C). Certain archetypes appear to be continental to an extent; for example, the Middle East and Oceania are showing a higher proportion of *Firmicutes* than other regions. In contrast to taxonomic variation, functional pathways were much more stable across continents, showing relatively slight variation in the abundance of high-level categories (Figure 2D). This pattern may also be due to the more limited range of pathway classes and their essential role in cellular function, in contrast to the much more wide-ranging taxonomic distributions examined across metagenomes. Classes of antimicrobial resistance were observed to vary by continent, as well as to occur in groups of taxonomically similar samples (Figure 2E) but were generally much sparser and more variable than the taxonomic gradients. We compared the distribution of pairwise distances between samples’ taxonomic profiles and their functional profiles (both equivalently normalized). Taxonomic profiles showed a mean pairwise Jensen-Shannon divergence (JSD) of 0.61, while pathways have a mean JSD of 0.099, which was significantly different (Welch’s t test, unequal variances, p < 2*e*16). This observation is consistent with data from the HMP, where the metabolic function varied less than taxonomic composition (Consortium et al., 2012; Lloyd-Price et al., 2017) within samples from a given body site.

### Microbial signatures reveal urban characteristics

**Figure 3:**
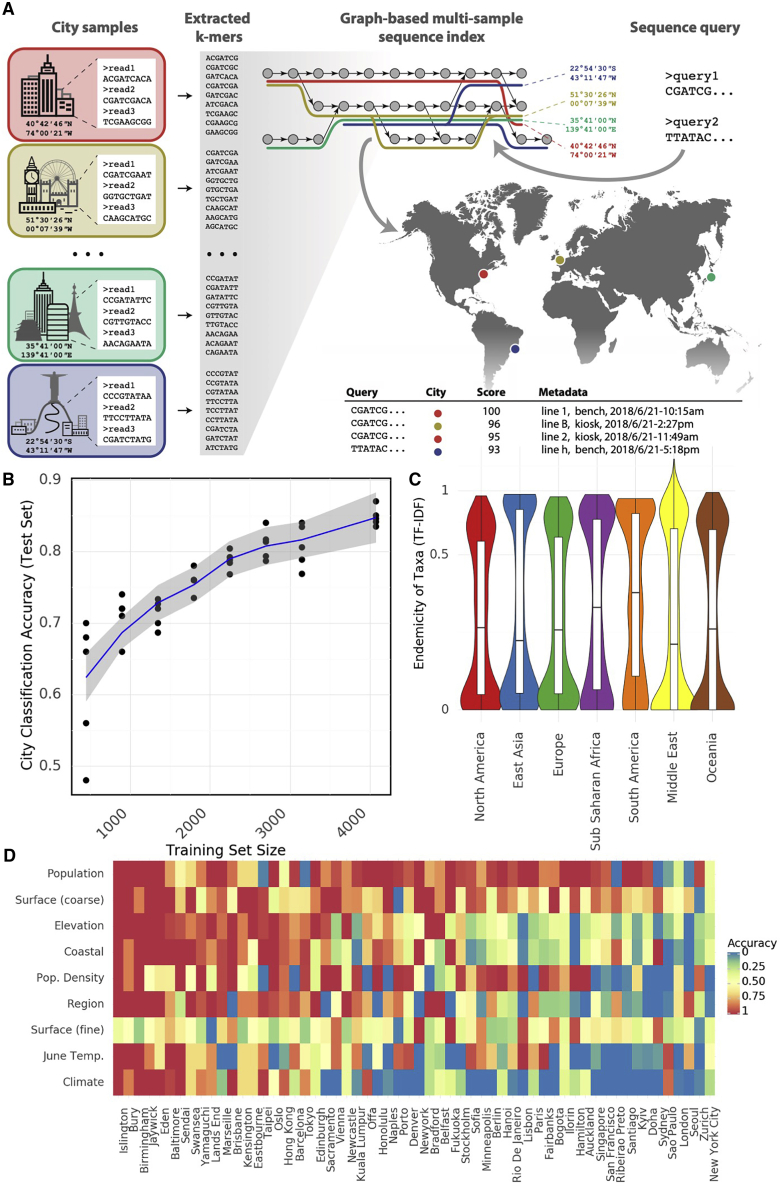
Microbial signatures (A) Schematic of GeoDNA representation generation. Raw sequences of individual samples for all cities are transformed into lists of unique k-mers (left). After filtration, the k-mers are assembled into a graph index database. Each k-mer is then associated with its respective city label and other informative metadata, such as geo-location and sampling information (top middle). Arbitrary input sequences (top right) can then be efficiently queried against the index, returning a ranked list of matching paths in the graph together with metadata and a score indicating the percentage of k-mer identity (bottom right). The geo-information of each sample is used to highlight the locations of samples that contain sequences identical or close to the queried sequence (middle right). (B) Classification accuracy of a random forest model for assigning city labels to samples as a function of the size of the training set. (C) Distribution of endemicity scores (term frequency inverse document frequency) for taxa in each region. (D) Prediction accuracy of a random forest model for a given feature (rows) in samples from a city (columns) that were not present in the training set. Rows and columns are sorted by average accuracy. Continuous features (e.g., population) were discretized. See also Figure S4.

To facilitate more straightforward mapping and comparison of sequences, we created GeoDNA and MetaGraph (https://metagraph.ethz.ch/search), a high-level web interface (Figure 3A) to search raw sequences against the MetaSUB dataset. Users can submit sequences to be processed against a k-mer graph-based representation of the MetaSUB data and other sequence databases (e.g., SRA). Query sequences are mapped to samples and collection metadata, and then a set of likely sample hits from around the world is returned to the user. This interface allows researchers to probe the diversity in this dataset and rapidly identify related genetic sequences, as well as the discovery of city-defining k-mers and sequences that might have forensic implications.

To test this idea of a sample’s predictive capacity for mapping to its city of origin, we trained a Random Forest classifier (RFC) from the taxonomic profile of each metagenome. Specifically, we trained an RFC with 100 estimators on 90% of the samples in our dataset and evaluated its classification accuracy on the remaining 10%. We repeated this procedure with multiple subsamples of our data at various sizes (with five replicates per size) to show how performance varied with the amount of input data (Figure 3B). The RFC achieved 88% on held-out data, which compares favorably to the 7.01% that would be achieved by a randomized classifier. Of note, we obtained similar results even with lower numbers of estimators (e.g., 10 estimators showed an accuracy of 78.9%). These results from our RFC demonstrate that city-specific taxonomic signatures and k-mers can be predictive for a sample’s origin.

**Figure S4:**
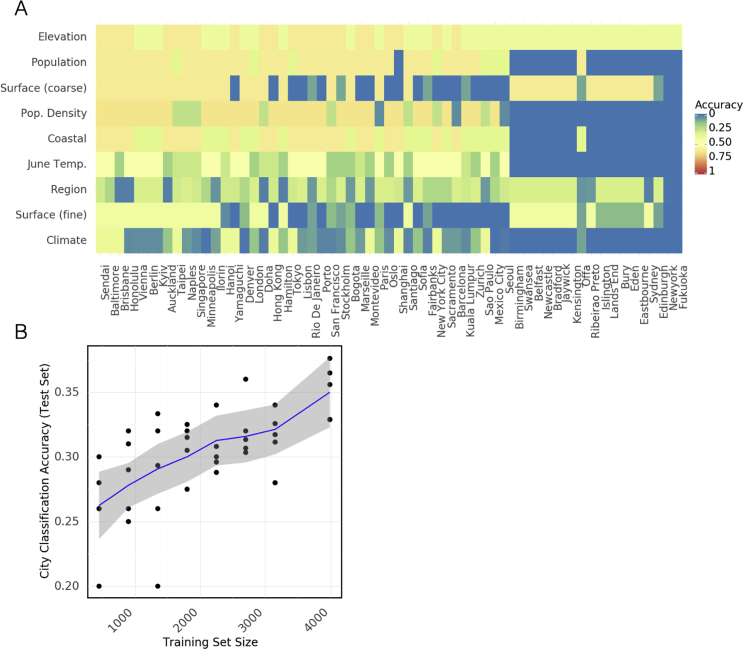
Microbial signatures in the urban environment, related to Figure 3 (A) Classification accuracy that would be achieved by a random model predicting features (rows) for held out cities (columns). (B) Classification accuracy of a random forest model predicting city labels for held out samples from antimicrobial resistance genes.

We next expanded our analysis of environmental taxonomic signatures to the prediction of features in cities not present in our training set, including population, surface material, elevation, proximity to the coast, population density, region, average June temperature, and Koppen climate classification. We trained an RFC to predict each feature based on all samples that were not taken from a given city, then used the relevant RFC to predict the feature for samples from the held-out city and recorded the classification accuracy (Figure 3D). While not all features and cities were equally predictable (in particular, features for several British cities were roughly similar and could be predicted effectively), in general, the predictions exceeded random chance by a significant margin (Figure S4A). The successful geographic classification of samples demonstrates distinct city-specific trends in the detected taxa and city metadata that may enable future forensic biogeographical capacities.

However, these city-specific taxa are not uniformly distributed across the world (Figure 3B). To quantify this “metagenome uniqueness” for each city, we developed a score to reflect how endemic a given taxon is within a city, which reflects the forensic usefulness of a taxon. We defined the endemicity score (ES) of a taxa as term-frequency inverse document frequency, where the “document” consists of samples from a group such as a city or region. This score is designed to simultaneously reflect the chance that a taxon would be useful to identify a given city. A high ES for a taxon in a city could be evidence of an evolutionary advantage in that city or neutral evolutionary drift, and the ES alone does not distinguish between the two. The distribution of ES shows a bimodal distribution for regions and cities (Figure 3C), with some outlier cities. Each region possesses a number of taxa with ES scores close to 1 and a slightly larger number close to 0 (note, ES is not bounded in [0, 1]). Some cities, such as Offa (Nigeria), host many taxa with high ES while others, such as Zurich (Switzerland), host fewer. High ES could indicate geographic sampling bias; however, some cities from well-sampled continents (e.g., Lisbon, Hong Kong) host many endemic species, suggesting that ES may indicate interchangeability and local niches of microbiome variation.

### Antimicrobial resistance genes form distinct clusters

**Figure 4:**
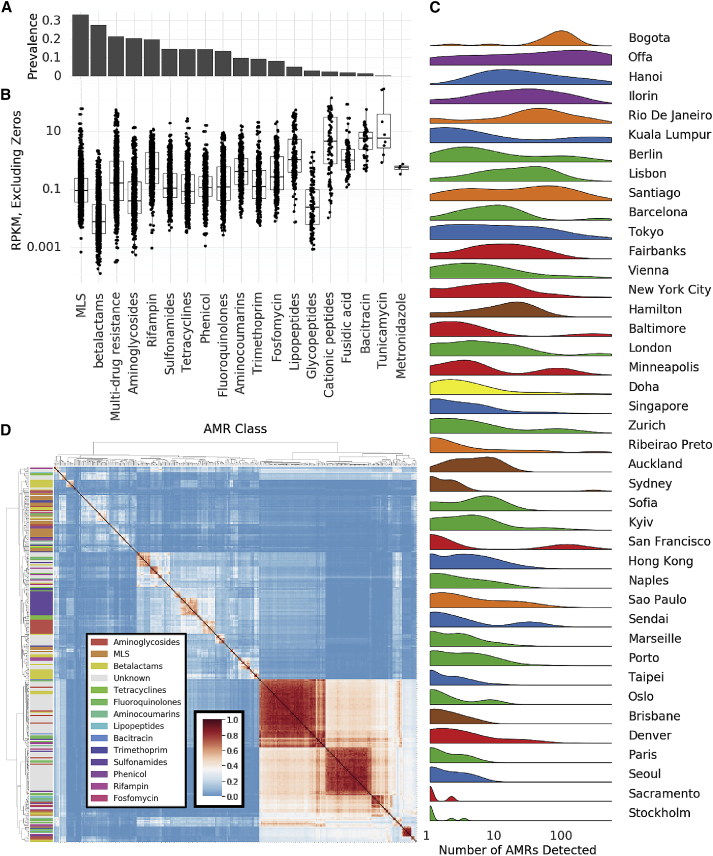
Antimicrobial resistance genes (A) Prevalence of AMR genes with resistance to particular drug classes. (B) Abundance of AMR gene classes when detected, by drug class. (C) Number of detected AMR genes by city. (D) Co-occurrence of AMR genes in samples (Jaccard index) annotated by drug class. See also Figure S5.

**Figure S5:**
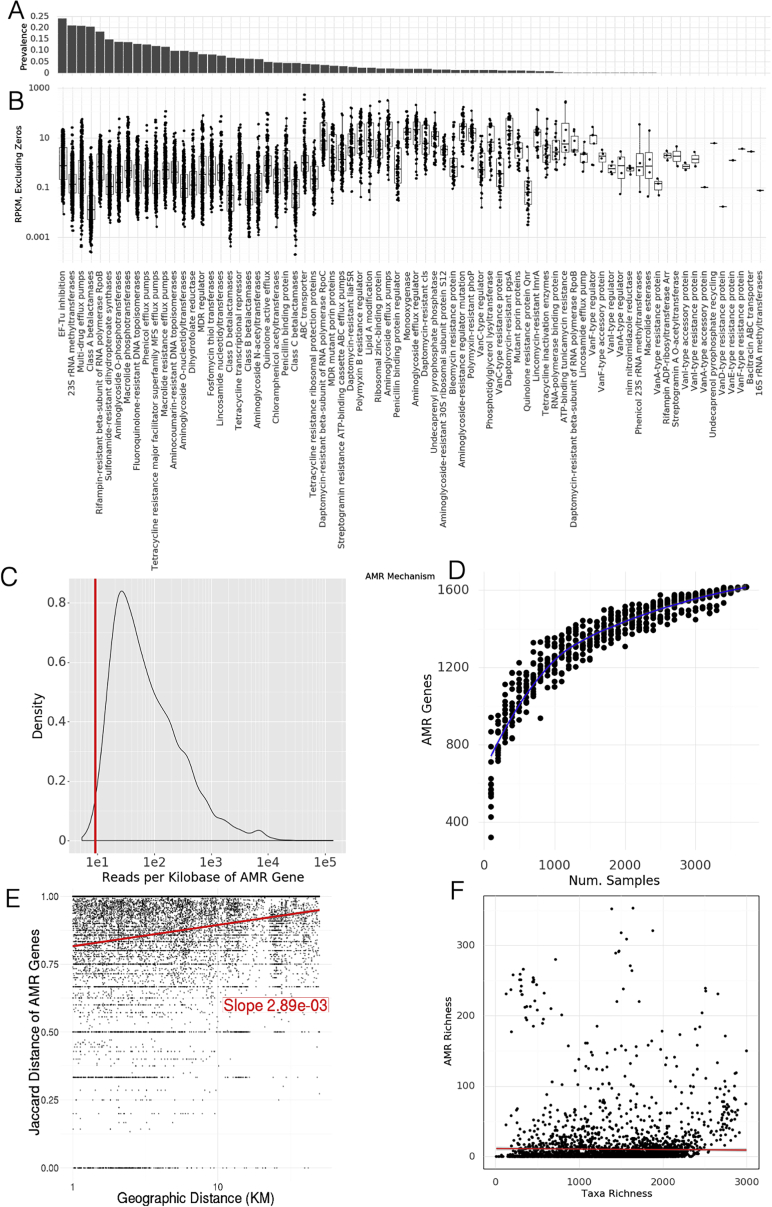
Antimicrobial resistance in the urban environment, related to Figure 4 (A) Prevalence of AMR genes with a particular resistance mechanism. (B) Abundance of AMR genes when categorized by resistance mechanism. (C) Distribution of reads per gene (normalized by kilobases of gene length) for AMR gene calls. The vertical red line indicates that 99% of AMR genes have more than 9.06 reads per kilobase and would still be called at a lower read depth. (D) Rarefaction analysis of antimicrobial resistance genes. Curve does not flatten suggesting we would identify more AMR genes with more samples. (E) Neighborhood effect. Jaccard distance of AMR genes weakly correlates with geographic distance within cities. (F) Relationship of the number of AMR genes (richness) to the number of species (richness) in each sample. No clear correlation is observed.

Quantification of antimicrobial diversity and AMRs are key components of global antibiotic stewardship. Yet, predicting antibiotic resistance from genetic sequences alone is challenging, and detection accuracy depends on the class of antibiotics (i.e., some AMR genes are associated with main metabolic pathways, while others are uniquely used to metabolize antibiotics). As a first step toward a global survey of antibiotic resistance in urban environments, we mapped reads to known antibiotic resistance genes, using the MegaRES ontology and alignment software. We quantified their relative abundance using reads/kilobase/million mapped reads (RPKM) for 20 classes of antibiotic resistance genes detected in our samples (Figures 4A and 4B). 2,210 samples had some sequences aligning to an AMR gene, but no consistent core set of AMR genes was identified. The most common classes of antibiotic resistance genes were for macrolides, lincosamides, streptogamines (MLS), and beta-lactams, yet the most common class of antibiotic resistance genes, MLS, was found in only 56% of the samples where AMR sequence was identified. We also quantified the likely mechanisms of identified antibiotic resistance genes. The three most prevalent resistance mechanisms are EF-Tu inhibition, 23S rRNA methyltransferases, and multi-drug efflux pumps. However, none of these are found in more than 25% of samples (abundance and prevalence of AMR mechanisms (Figures S5A and S5B).

Indeed, antibiotic resistance genes were universally in low abundance compared to functional genes, with RPKM values for resistance classes typically ranging from 0.1 to 1 compared to values of 10 to 100 for typical housekeeping genes (AMR classes contain many genes, so RPKM values may be lower than they would be for individual genes). Despite the low abundance of the genes themselves, some samples contained sequences from hundreds of distinct AMR genes. Clusters of high AMR diversity were not evenly distributed across cities (Figure 4C). Some cities had more resistance genes identified on average (15–20×) than others (e.g., Bogota), while other cities had bimodal distributions (e.g., San Francisco); some samples had hundreds of genes, while others were very few. We note that 99% of the cases where we detected AMR genes showed an average depth of 2.7×, indicating that our overall global distribution would not dramatically change with altered read depth (Figure S5E).

Since taxa could be used to classify a sample’s city of origin, we next examined if AMR genes exhibited the same stratification. A random forest model was trained (as above) to predict city classification based on the mapped antimicrobial resistance genes. While this model achieved 37.6% accuracy on held out test data (Figure S4B), showing that it is better than random chance (7.0%), the AMR profile was much less accurate than the taxonomic predictor (88.0%). Since AMR genes are more likely to be mobile, this is not surprising and likely indicates that they represent weaker (but possible) city-specific signatures.

Prior studies have shown that numerous AMR genes can be carried on a single plasmid, and ecological competition may cause multiple taxa in the same sample to develop antimicrobial resistance, but little is known in urban environments. To examine these phenomena, we identified clusters of AMR genes that co-occurred in the same samples (Figure 4D). We measured the Jaccard distance between all pairs of AMR genes found in at least 1% of samples and performed agglomerative clustering on the resulting distance matrix. We identified three large clusters of genes and numerous smaller clusters. Of note, these clusters often consist of genes from multiple classes of resistance, and the large clusters contain far more genes than are typically found on plasmids.

Next, we performed a rarefaction analysis on the set of all resistance genes in the dataset, which we call the “panresistome” (Figure S5D). Similar to the rate of detected species, the panresistome also shows an open slope with an expected rate of discovery of 1 new AMR gene per 10 samples. Given that AMR gene databases are rapidly expanding, and that no AMR genes were found in some samples, it is likely that future analyses will identify many more resistance genes in these data. Additionally, AMR genes showed a “neighborhood” effect within samples that are geographically proximal, analogous to the effect was seen for taxonomic composition (Figure S5C). Excluding samples where no AMR genes were detected, the Jaccard distance between sets of AMR genes increases with distance for pairs of samples in the same city. As with taxonomic composition, the overall effect is weak and noisy but nonetheless significant.

### Widespread observation of biology not in reference databases

**Figure 5:**
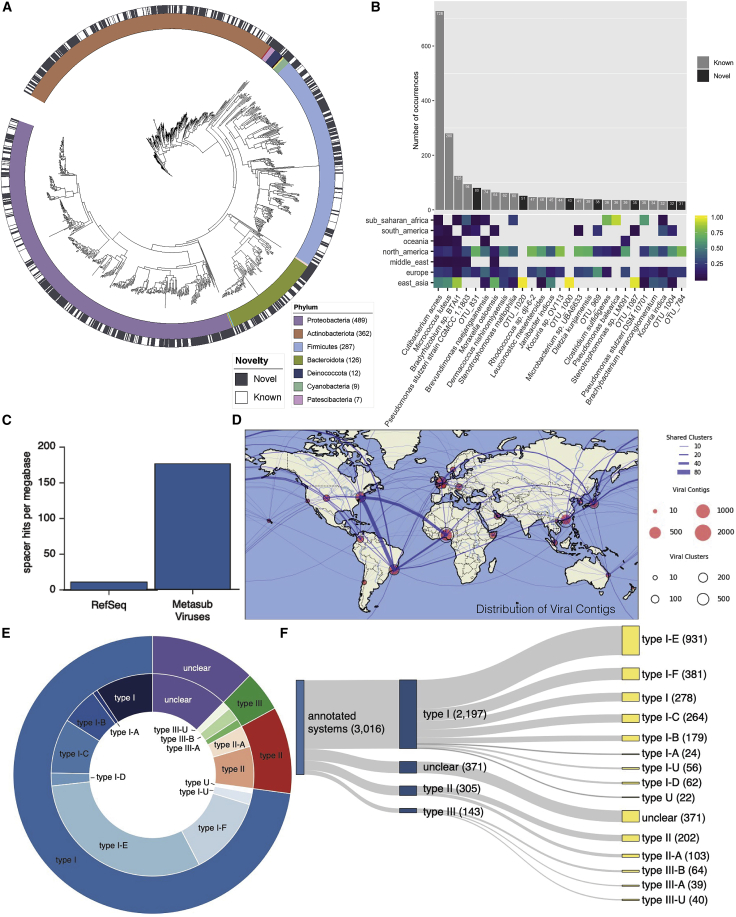
Newly observed genetic sequences (A) Taxonomic tree for metagenome-assembled genomes (MAGs) found in the MetaSUB data. The outer black and white ring indicate if the MAG matches a known species, and the inner ring indicates phyla of the MAG. (B) Top: the number of samples where the most prevalent MAGs were found. Bottom: the regional breakdown of samples where the MAG was found. (C) Mapping rate of CRISPR spacers from MetaSUB data to viral genomes in RefSeq and viral genomes found in MetaSUB data. (D) Geographic distribution of viral genomes found in MetaSUB data. (E and F) Fractional breakdowns of identifiable CRISPR systems found in the MetaSUB data.

To examine these samples for large genetic elements, we created metagenome-assembled genomes (MAGs) with metaSPAdes to look for viral, bacterial, and archaeal genomes and for CRISPR arrays (see assembly methods). These MAGs comprised 1,304 total high-quality genomes, of which 748 did not match any known reference genome within 95% ANI. 1,302 of the genomes were classified as bacteria and 2 as archaea. Bacterial genomes came predominantly from four phyla: the Proteobacteria, Actinobacteria, Firmicutes, and Bacteroidota. Bacterial genomes that did not match any reference were evenly spread across these phyla (Figure 5A), and assembled bacterial genomes were often identified in multiple samples. Several of the most prevalent bacterial genomes were species with no known reference genome with >95% average nucleotide identity (Figure 5B).

Some assembled genomes showed regional specificity, while others were globally distributed. Overall, the taxonomic composition of identifiable genomes roughly matched the composition of the core urban microbiome (see a core urban microbiome centers global diversity), with the number of identified bacterial MAGs somewhat related to read depth (number of reads correlated with the number of OTUs in sample with *R* = 0.4, p < 2*e* 16 Pearson’s correlation), indicating additional sampling and sequencing will continue to discover more MAGs that do not match known reference genomes. Bacterial MAGs were roughly evenly distributed geographically, with the notable exception of Offa, Nigeria, which had dramatically more bacterial species than other cities that did not match references.

We then examined the assembled contigs for viruses using Joint Genome Institute’s (JGI’s) uncultivated viral genomes (UViGs) mapping method (Paez-Espino et al., 2019). This analysis revealed a set of 16,584 total UViGs. Taxonomic analysis of the predicted UViGS yielded 2,009 viral clusters, containing a total of 6,979 UViGs and 9,605 singleton UViGs for a total of 11,614 predicted viral species. Predicted viral species from samples collected within 10, 100, and 1,000 km of one another were agglomerated to examine their planetary distribution at different scales (Figure 5B). At any scale, most viral clusters appear to be weakly cosmopolitan; the majority of their members are found at or near one location, with a few exceptions.

We compared the MAG-derived viruses to known viral sequences in the Integrated Microbial Genome and Viral database (IMG/VR) at JGI, which contains viral genomes from isolates, a curated set of prophages, and 730,000 viral MAGs from other studies. Of the 11,614 species discovered in the MetaSUB MAGs, 94.1% did not match any viral sequence in IMG/VR (Paez-Espino et al., 2019) at the species level for a total of 10,928 viruses that did not match known species. We note that this number was obtained using a conservative pipeline (99.6% precision) and corresponded well with our identified CRISPR arrays (below). This suggests that urban microbiomes contain a large, untapped amount of viral diversity not previously observed in other environments.

Next, we attempted to identify possible bacterial and eukaryotic hosts for our predicted viral MAGs. For the 686 species with similar sequences in IMG/VR, we projected known host information onto 2,064 MetaSUB viral MAGs. Additionally, we used CRISPR-Cas spacer matches in the Integrated Microbial Genomes and Microbiomes (IMG/M) system to assign possible hosts to a further 1,915 predicted viral species. Finally, we used a database of 20 million metagenome-derived CRISPR spacers to provide further rough taxonomic assignments. Our predicted viral hosts aligned with our taxonomic profiles; 41% of species in the core microbiome (see a core urban microbiome centers global diversity) had predicted viral-host interactions. Many of our viral MAGs were found in multiple locations (Figure 5D). Many viruses were found in South America, North America, and Africa, and viral MAGs in Japan often corresponded to those in Europe and North America.

From these MAGs, we identified 838,532 CRISPR arrays, of which 3,245 could be annotated for specific CRISPR systems. The annotated CRISPR arrays were mostly type 1-E and 1-F, but a number of type II and III systems were identified as well (Figures 5E and 5F), and some arrays had unclear (ambiguous) type assignment. Critically, when we aligned spacers to both our viral MAGs and all viral sequences in RefSeq, the spacers in our identified CRISPR arrays closely matched our predicted MAG-derived viruses. Moreover, while the total fraction of spacers that could be mapped to our virus-containing MAGS and RefSeq was similar (32.2% to our data versus 36.8% for RefSeq), the mapping rate to our viral MAGs dramatically exceeded the mapping rate to RefSeq (Figure 5C), which provides additional evidence supporting the veracity of urban viruses.

## Discussion

MetaSUB is a global network of scientists and clinicians developing knowledge of urban microbiomes by studying mass transit systems, the built environment, and hospitals. We collected and sequenced 4,728 samples from 60 cities worldwide (Table 1; Table S1), constituting the first large-scale metagenomic study of the urban metagenome. We conclude that there is a consistent urban microbiome core (Figures 1 and 2), which is supplemented by geographic variation (Figure 2) and microbial signatures based on the specific attributes of a city (Figure 3). Our data also show that taxa remain to be discovered in these and future data (Figure 5), environmental factors (e.g., climate) significantly affect the microbial variation, and sequences associated with AMR genes are globally widespread but not necessarily abundant (Figure 4). In addition to these results, we present several ways to access and analyze our data including interactive web-based visualizations, search tools over raw sequence data, and high-level interfaces to computationally access results.

Together, these data suggest that urban microbiomes should be treated as ecologically distinct from both surrounding soil microbiomes and human commensal microbiomes. Though these microbiomes undoubtedly interact, they nonetheless represent distinct ecological niches with different genetic profiles. While our metadata covariates were associated with the principal variation in our samples, they do not explain a large proportion of the observed variance. It remains to be determined whether the variation is essentially a stochastic process or if a deeper analysis of our covariates proves more fruitful. In particular, analysis of cities’ greenspace, tourism, and waste management systems may be fruitful to explain variation; a study by Reese et al., (2016) found that urban stress could impact microbial composition. We have observed that less important PCs (roughly PCs 10–100) are generally less associated with metadata covariates but that PCs 1–3 do not adequately describe the data alone. This is a pattern that was observed in the human microbiome project as well, where minor PCs (such as our Figure 2B) were required to separate samples from closely related body sites.

Much of the urban microbiome likely represents previously unobserved diversity, as our samples contain a significant proportion of unclassified DNA. This finding is comparable to many other metagenomic and microbiome studies including other work is done in subway environments (Afshinnekoo et al., 2015; Hsu et al., 2016), airborne microbiomes (Yooseph et al., 2013), work done by the Earth Microbiome Project (Thompson et al., 2017), and others. As noted in Figure 1, more sensitive alignment methodology only marginally increases the proportion of classified DNA. We consider the DNA that would not be classified by a sensitive technique to be truly unclassified DNA and postulate that it may derive from genes or species not in reference databases. Given that our samples did not closely resemble human commensal microbiomes or soil samples, it is possible this represents DNA sequences specific to the urban environment.

The fraction of predicted viral sequences that belonged to previously unobserved taxa was particularly high in our study (94.1%); however, taxonomic associations of these viruses to observed microbial hosts and associations with novel CRISPR sequences suggest these results are not spurious. The discovery of more taxa not in reference databases may help to reduce the large fraction of DNA that cannot currently be classified. Our data do not support the presence of any viruses in the core microbiome. However, this cannot be excluded and should be thoroughly addressed in the future with more in-depth sequencing, sampling/extraction techniques, or long-read technologies.

Many of the identified taxa are frequently implicated as infectious agents in a clinical setting including specific *Staphylococcus*, *Streptococcus*, *Corynebacterium*, *Klebsiella*, and *Enterobacter* species. However, there is no indication that the species identified in the urban environments are pathogenic, and further in-depth studies are necessary to determine the clinical impact of urban microbiomes. This includes microbial culture studies, explicitly searching for virulence factors and performing strain-level characterization to determine biological functions carried by specific populations. Seasonal variation also remains open to study as the majority of the samples collected here were from two global city sampling days (June 21, 2016, and June 21, 2017). Further studies, some generating novel data, will need to explore whether the core microbiome shifts over the course of the year, with a particular interest in the role of the microbiome in flu transmission (Cáliz et al., 2018; Korownyk et al., 2018).

The coronavirus disease 2019 crisis has thrown the need for broad microbial surveillance into sharp relief. Microbial genetic mapping of urban environments will give public health officials tools to assess risk, map outbreaks, and genetically characterize problematic species. This study identifies a large number of viruses in the environment as well as antimicrobial resistance genes in bacteria, but they are only DNA based. Future shotgun RNA studies (metatranscriptomics) and targeted RNA viral studies that build on top of this infrastructure represent an important starting point for tracking and potentially mitigating future epidemics.

As metagenomics and next-generation sequencing becomes more and more available for clinical (Wilson et al., 2019) and municipal use (Hendriksen et al., 2019), it is essential to contextualize the AMR markers or presence of species and strains within a global and longitudinal context. We observed that the microbial profile of cities can slightly shift year to year and that this may become a more pronounced effect over longer time frames. The most common AMR genes were found for two classes of antibiotics: MLS and beta-lactams. Both of these are critical groups of antibiotics used to treat upper respiratory, skin, soft tissue, and sexually transmitted infections and a wide array of other infections. Antimicrobial resistance genes are thought to spread from a variety of sources including hospitals, agriculture, and water (Bougnom and Piddock, 2017; Klein et al., 2018). The antimicrobial classes particularly impacted by resistance include beta-lactams, glycopeptides, and fluoroquinolones (Rice, 2012), all of which we found antimicrobial resistance genes across our samples.

We found that there was an uneven distribution of AMR genes across cities and that fewer AMR genes were identified in samples from Oceania and the Middle East. This could be the result of different levels of antibiotic use, differences in the urban geography between cities, or reflect the background microbiome in different places in the world. Techniques to estimate antibiotic resistance from sequencing data remain an area of intense research as certain classes of AMR gene (i.e., fluoroquinolones) are sensitive to small mutations, and methodological improvements may refine our results. A companion study to this paper by Chng et al. (2020) has examined the spread of AMRs in hospital settings. Further research is needed to explore AMR genes fully in the urban environment, especially in medical environments, including cultural studies that directly measure the phenotype of resistance.

In summary, this study presents the first genetic atlas of urban and mass-transit metagenomics from across the world. By facilitating large-scale epidemiological comparisons, it is a first critical step toward quantifying the distribution, types, and dynamics of environmental microbiomes, providing requisite data for tracking changes in ecology or virulence. As more datasets emerge from rural and suburban areas with livestock and farms, sewage from cities (Fresia et al., 2019; Joseph et al., 2019), and other public sources of AMR genes, a new international AMR mapping paradigm is possible. Ideally, these data are components of a global sentinel monitoring network of sequencers that tracks AMR and other microbial changes (Singer et al., 2016; Thanner et al., 2016), which can also help with clinical interpretation and risk stratification (Afshinnekoo et al., 2017; Gardy and Loman, 2018; Ladner et al., 2019). Indeed, a continually updated, global microbial genetic atlas has the potential to aid physicians, public health departments, government officials, and scientists in tracing, diagnosing, and predicting epidemiological risks and trends. This, in turn, enables data-driven policy and medical decisions in cities around the world, with the sequencing data simultaneously providing a constant fountain of discovery for new microbial biology.

### Limitations of the study

There are three key limitations to this study. First, this study exclusively measured DNA, meaning RNA viruses would be excluded, as would evidence of transcriptional activity from Bacteria and Archaea. Second, this study is unable to identify a large proportion of DNA collected. This is at least partly due to the highly novel nature of urban microbiomes, and as more data are generated, this proportion could be improved. Third, AMR genes are often difficult to distinguish from similar genes that do not confer resistance (though we have removed genes that require SNP-level verification), so our results likely have a degree of noise.

## STAR★Methods

### Key resources table

**Table:** 

REAGENT or RESOURCE	SOURCE	IDENTIFIER
**Bacterial and virus strains**		

ZymoBiOMICS Microbial Community standard	Zymo Research	Catalog #D6300
ZymoBIOMICS Microbial Community DNA Standard	Zymo Research	Catalog #D6305

**Biological samples**		

Environmental samples from urban and built-in structures	Participating Consortium members	N/A

**Critical commercial assays**		

QIAGEN QIAseq FX DNA Library Kit	QIAGEN	ID: 180475
Promega DNA extraction Maxwell kit Blood	Promega	AS1400
Promega DNA extraction Maxwell kit Buccal Swab	Promega	AS1640
Zymo DNA/RNA shield	Zymo Research	R1100-250
MoBio PowerSoilR©DNA Isolation Kit	MoBIO	Cat.:12888-100
Agencourt AMPure XP	Beckmann Coulter	Cat.:A63881
Qubit® dsDNA HS Assay	Thermofisher	Q32854
QuantiFluor® ONE dsDNA System	Promega	E4871
Nextera Flex (Now known as Illumina DNA Prep)	Illumina	20018705
Nextera DNA CD Indexes	Illumina	20018708

**Deposited data**		

NCBI/RefSeq Microbial ca. March 2017	NCBI	https://www.ncbi.nlm.nih.gov/refseq/
Hg38 with Alternate Contigs	UCLA	https://hgdownload.cse.ucsc.edu/goldenPath/hg38
Human Microbiome Project	Human Microbiome Project Consortium, 2012	https://www.hmpdacc.org/hmp/resources/download.php
Microbe Directory	Shaaban et al., 2018	https://microbe.directory
UniRef90	Suzek et al., 2007	https://www.uniprot.org/downloads
Integrated Gut Genomes v1.0	Nayfach et al., 2019	https://github.com/snayfach/IGGdb
Genome Taxonomy Database	Parks et al., 2018	https://gtdb.ecogenomic.org/downloads
MetaSUB Sequencing Data	This paper	https://pngb.io/metasub-2021

**Software and algorithms**		

AdapterRemoval v2.17	Schubert et al., 2016	https://github.com/mikkelschubert/adapterremoval
Bowtie2 v2.3.0	Langmead and Salzberg, 2013	https://sourceforge.net/projects/bowtie-bio/files/bowtie2/2.3.0/
BLASTn	Altschul et al., 1990	https://ftp.ncbi.nlm.nih.gov/blast/executables/blast+/LATEST/
KrakenUniq v0.3.2	Breitwieser et al., 2018	https://github.com/fbreitwieser/krakenuniq
MASH v2.1.1	Ondov et al., 2016	https://github.com/marbl/Mash
HUMAnN2	Franzosa et al., 2018	https://pypi.org/project/humann2/
DIAMOND v0.8.36	Buchfink et al., 2015	https://github.com/bbuchfink/diamond
metaSPAdes v3.8.1	Nurk et al., 2017	https://github.com/ablab/spades/releases/tag/v3.8.1
MegaRes v1.0.1	Lakin et al., 2017	https://megares.meglab.org/download/index.php
MetaBAT2 v2.12.1	Kang et al., 2019	https://anaconda.org/ursky/metabat2
CheckM v1.0.13	Parks et al., 2015	https://github.com/Ecogenomics/CheckM
dnadiff v1.3	Kurtz et al. 2004	https://github.com/mummer4/mummer
GTDB-Tk v1.0.2	Chaumeil et al., 2019	https://github.com/jianshu93/GTDB_Tk
FastTree v2.1.10	Price et al., 2010	https://anaconda.org/bioconda/fasttree
iTOL v5.5	Letunic and Bork 2019	https://itol.embl.de/
CRISPRCasFinder	Couvin et al., 2018	https://github.com/dcouvin/CRISPRCasFinder
SciPy	Virtanen et al., 2020	https://www.scipy.org/
dendextend v1.12.0	Galili 2015	https://github.com/cran/dendextend
MUMmer v3.23	Kurtz et al., 2004	https://github.com/mummer4/mummer
ResistomeAnalyzer (commit 15a52dd)	Lakin et al., 2017	https://github.com/cdeanj/resistomeanalyzer
MetaSUB Core Analysis Pipeline	Danko and Mason 2020	https://github.com/MetaSUB/CAP2
CAPalyzer	Danko and Mason 2020	https://github.com/dcdanko/capalyzer
Figure Generation Code	This paper	https://github.com/MetaSUB/main_paper_figures

**Other**		

Copan Liquid Amies Elution Swab	Copan Diagnostic	Cat.:480C
Isohelix Swabs	Isohelix	Cat.:MS-02
2D Thermo Scientific Matrix	Thermo Scientific	3741-WP1D-BR
ZR BashingBead Lysis Tubes (0.1 & 0.5 mm)	Zymo Research	Cat# S6012-50

### Resource availability

#### Lead contact

Further information and requests for resources and reagents should be directed to and will be fulfilled by Christopher Mason (chm2042@med.cornell.edu).

#### Materials availability

This study did not generate any new.

#### Data and code availability

##### Materials, Methods, and Open-Source Code

To make our study fully reproducible, we released an open-source version-controlled pipeline called the MetaSUB Core Analysis Pipeline (CAP) (Danko and Mason, 2020). This pipeline includes all steps from extracting data from raw sequence FASTQ files to producing refined results like taxonomic and functional profiles. Every tool in the CAP is open source with a permissive license. The CAP is available as a docker container for easier installation in some instances, and all databases used in the CAP are available for public download. The CAP is versioned and includes all necessary databases, allowing researchers to replicate results and figures.

The MetaSUB dataset and CAP are built and organized for full accessibility to other researchers. This is consistent with the concept of Open Science. Specifically, we built our study with the FAIR principles in mind: Findable, Accessible, Interoperable, and Reusable. To make our results more reproducible and accessible, we have developed a program to merge the CAP’s output into a condensed data-packet. This data packet contains results as a series of Tidy-style data tables with descriptions. The advantage of this set-up is that result tables for an entire dataset can be parsed with a single command in most high level analysis languages like Python and R. This package also contains Python utilities for parsing and analyzing data packets which streamline most of the boilerplate tasks of data analysis. All development of the CAP and data packet builder (Capalyzer) package is open source and permissively licensed.

In addition to general-purpose data analysis tools, essentially all analysis in this paper is available as a series of Jupyter notebooks. These notebooks allow researchers to reproduce our results, build upon our results in different contexts, and better understand precisely how we arrived at our conclusions. By providing the exact source used to generate our analyses and figures, users can quickly incorporate new data or correct any bugs.

For less technical purposes, we also provide web-based interactive visualizations of our dataset (typically broken into city-specific groups). These visualizations are intended to provide a quick reference for major results as well as an exploratory platform for generating novel hypotheses and serendipitous discovery. The web platform used, MetaGenScope, is open source, permissively licensed, and can be run on a moderately powerful machine (though its output relies on results from the MetaSUB CAP).

Our hope is that by making our dataset open and easily accessible to other researchers the scientific community can more rapidly generate and test hypotheses. One of the core goals of the MetaSUB consortium is to build a dataset that benefits public health. As the project develops, we want to make our data easy to use and access for clinicians and public health officials who may not have computational or microbiological expertise. We intend to continue to build tooling that supports these goals.

##### CAMDA

Since 2017, MetaSUB has partnered with the Critical Assessment of Massive Data Analysis (CAMDA) camda.info, a whole conference track at the Intelligent Systems for Molecular Biology (ISMB) Conference. At this venue, a subset of the MetaSUB data was released to the CAMDA community in the form of an annual challenge addressing the issue of geographically locating samples: ‘The MetaSUB Inter-City Challenge’ in 2017 and ‘The MetaSUB Forensics Challenge’ in 2018 and 2019. In the latter challenge the MetaSUB data has been complemented by data from EMP (Thompson et al., 2017) and other studies (Delgado-Baquerizo et al., 2018; Hsu et al., 2016). This Open Science approach of CAMDA has generated multiple interesting results and concepts relating to urban microbiomics, resulting in several publications https://biologydirect.biomedcentral.com/articles/collections/camdaproc as well as perspective manuscript about moving toward metagenomics in the intelligence community (Mason-Buck et al., 2020). The partnership is continued in 2020 with ‘The Metagenomic Geolocation Challenge’ where the MetaSUB data has been complemented by the climate/weather data in order to construct multi-source microbiome fingerprints and predict the originating ecological niche of the sample.

##### Accessions and data access

All data from this study including data tables that resulted from analyses may be found at https://pngb.io/metasub-2021. Additionally, raw sequencing reads are uploaded to the SRA and may be found under the accession SRA ID: PRJNA732392.

### Method details

#### Sample collection and preparation

To obtain a comprehensive picture of microbial communities within a sample, it is essential to choose a sampling method which absorbs and preserves biological materials during sampling, transport and storage until DNA extraction. The effectiveness of a swab may be influenced by a number of factors, most importantly the material of the swab tip which can affect the rate at which bacteria are collected during the sampling process. Furthermore, the design of the transport tube as well as the DNA preserving liquids can affect the integrity of the material during transport. Finally, the amount of background contamination identified for different products should be taken into account.

##### Sampling materials

Surface samples were collected and preserved using a flocked swab with a storage tube containing a buffer that is optimized for DNA preservation. Two different sets of materials were used for collection in 2016 and 2017.

In the first method of sample collection used a Copan Liquid Amies Elution Swab (ESwab, Copan Diagnostics, Cat.: 480C) paired with a 1mL of Liquid Amies in a plastic, screw cap tube, hereafter referred to as a ‘Copan swab’. The Amies transport medium maintains the sample at pH 7.0 0.5 and contains sodium thioglycolate as well as calcium, magnesium, sodium, and potassium salts to control the permeability of bacterial cells. Once the surface was sampled, the swab was immediately placed into the collection tube and stored in a −80C freezer once returned to the laboratory.

The second method used an individually wrapped Isohelix Buccal Mini Swab (MS Mini DNA/RNA Swab, Isohelix, Cat.: MS-02) paired with a barcoded storage tubes (2D Matrix V-Bottom ScrewTop Tubes, Thermo Scientific, Cat.: 3741-WP1D-BR/1.0mL), hereafter referred to as ‘matrix tubes’, prefilled with 400*μ*l of a transport and storage medium suitable for both DNA and RNA (DNA/RNA Shield, Zymo Research, Cat.: R1100), hereafter referred to as ’Zymo Shield’. Once the surface was sampled, the swab was immediately placed into a matrix tube containing Zymo Shield and stored in a −80C freezer until DNA extraction.

We assessed the absorption strength of both the Copan and Isohelix swabs for various biological and surface materials encountered when sampling metro stations. A single surface was selected for a designated sampling area to test the absorption strength. Both swabs were moistened by submerging the swab for a few seconds in their preservative media. The area was then swabbed for 3 min, covering the selected surface. By moistening the swab prior to sampling, the swab matrix would take up more microflora already saturated with the transport medium.

##### Sampling protocol

A standard operating procedure (SOP) was developed for the sample collection to be followed by all members of the MetaSUB consortium participating in CSD, and adapted from earlier work by Afshinnekoo et al. (2015). The aim was to standardize as much of the sampling procedure in order to ensure high quality control across the various cities and sampling teams. Thus, it was recommended that teams collect samples from high contact surfaces found in most metro and transit stations and systems around the world, including ticket kiosks, turnstiles, railings, and seats or benches. Some cities had to adapt the sampling approach to better reflect their city. For example, in cases where a city did not have a subway system, the most common form of public transit was studied instead. While variation in the types of surfaces being sampled were allowed, modifications to the sampling procedure itself were not. Moreover, a number of metadata were recorded for each sample during the process of collection to ensure as much contextual information as possible was captured. Each city developed their own sampling and submitted them for review before sampling kits were sent to them in order to ensure consistency across the various sites.

All principal investigators and MetaSUB city leaders were trained in the sampling protocol and this training was further disseminated to the respective sampling teams to ensure consistent and quality control sampling. Each participant was instructed to don disposable latex or nitrile gloves prior to sample collection. The swab was dipped in the preservative medium for approximately 2 s before the swab was firmly dragged across the surface, using both sides and using different angles, for a total of 3 min to ensure highest yield. Any other important notes or observations could be added to the metadata for each sample.

A sampling protocol video overview is included in the Supplemental information.

##### Process controls

To assess the quality of our sampling procedure, we created multiple controlled scenarios. As a positive laboratory control, a Copan swab was introduced into a sterile urine cup with 30*μ*l of a well-defined, accurately characterized microbial reference sample (ZymoBIOMICS Microbial Community Standard, Zymo Research, Cat.: D6300). A negative control was made by adding 50*μ*l of the final resuspension buffer from the DNA isolation step into a sterile urine cup before introducing a Copan swab. Furthermore, a laboratory workbench was swabbed using our sampling procedure both before and after it was cleaned with a 10% bleach solution. To detect background contamination due to biological material in the air in sample areas, a dampened Copan swab was held in the air for approximately 3 min. Finally, to ensure there was no contamination could be due to the consumables we procured prior to sampling, we also swabbed, in triplicate, the interior of a flow hood that had been sterilized with 10% bleach before wiping down with ethanol and irradiating with ultraviolet light.

##### Metadata collection and aggregation

Metadata from individual cities was collected from a standardized form and set of data fields. The principal fields collected were the location of sampling, the material of the object being sampled, the type of object being sampled, the elevation above or below sea level, and the station or line where the sample was collected. However, several cities were unable to use the provided software application for various reasons, and instead submitted their metadata as separate spreadsheets that could be added to the data repository. Additionally, certain metadata features, such as those related to sequencing and quality control, were added after initial sample collection. To collate various metadata sources, we built a publicly available repository on Pangea (https://pngb.io/metasub-2021) which assembled a large master spreadsheet with consistent sample universally unique identifiers (UUID). After assembling the originally collected data attributes we added normalized attributes based on the original metadata to account for surface material, control status, and features of individual cities. A full description of ontologies used is provided as part of the collating program.

#### DNA extraction, library preparation, and sequencing

Samples stored at −80C were allowed to thaw to room temperature before performing a DNA extraction suitable to the transport and preservation medium used with the Copan swabs and Isohelix swabs in 2016 and 2017, respectively. Initially, Copan swabs in liquid Amies were processed using the PowerSoil DNA Isolation Kit (MoBio, Cat.: 12888-100), while Isohelix swabs were processed using the ZymoBIOMICS 96 MagBead DNA Kit (Zymo Research, Cat.: D4308). Additional automation of sample processing for nucleic acid extraction using the Maxwell RSC Instrument (Promega, Cat.: AS4500) began in 2017 using the Maxwell RSC Buccal Swab Kit (Promega, Cat.: AS1640).

##### DNA extraction from copan swabs

After spinning down the tubes containing the Copan swab in Amies at 300rpm for 1 min, the swab pad was transferred to a MoBio PowerBead Tube containing beads using sterile scissors, which we sterilized with 70% ethanol before passing them through a flame. The remaining 400-500*μ*l of Amies solution was transferred into an Eppendorf tube and centrifuged at high speed to collect bacteria and debris into a pellet. Once resuspended into a small volume of Amies, the pellet was transferred to the same MoBio PowerBead Tube as its corresponding Copan swab. The MoBio PowerSoil DNA Isolation Kit was used according to manufacturer’s instructions with the exception of the following modifications: both the swab and corresponding pellet were resuspended in 135*μ*l of the C1 buffer. Sample homogenization was performed using either the TissueLyser II (QIAGEN, Cat.: 85300) with 2 cycles of 3 min at 30Hz (https://bit.ly/3ub9tap) or using a Vortex-Genie 2 adaptor for 1.5 to 2mL tubes (Vortex Adaptor for 24 tubes, QIAGEN, Cat.: 13000-V1-24) at maximum speed for 10 min. The sequencing centers in Stockholm and Shanghai used different procedures for homogenization. Stockholm used a method based on MPI FASTPREP, while Shanghai added 0.6 g of 100-micron zirconium-silica beads to 2ml tubes containing the swab pad and the media, followed by bead beating for 1 min. Following the MoBio protocol, the eluted samples were additionally purified by introducing 1.8X of Agencourt AMPure XPmagnetic beads (Beckman Coulter, Cat.:A63881), allowed to incubate at 25C for 15 min, and then placed on an Invitrogen magnetic separation rack (MagnaRack) for 5 min. A wash step using 700*μ*l of 80% ethanol was added the samples while they remained on the MagnaRack before allowing the samples to dry. The resulting purified samples were eluted into 12*μ*l - 50*μ*l of buffer. Subsequently, DNA was quantified using a Qubit 2.0 fluorometer and (dsDNA HS Assay Kit, Invitrogen, Cat.: Q32854).

##### DNA Extraction from Isohelix Swabs

The entire 400*μ*l volume of Zymo Shield, along with the Isohelix swab head, were transferred into a new tube containing a 0.6mL dry volume of 0.5mm and 0.1mm lysis matrix (BashingBead Lysis Tubes, Zymo Research, Cat.: S6012-50), as well as an additional volume of 600*μ*l of Zymo Shield. Mechanical lysis using bead beating was performed on 18 samples at a time using a Vortex-Genie 2 adaptor at maximum power for 40 min. A 400*μ*l volume of the resulting lysate in each tube was transferred into a separate well of a deep-well storage plate (Nunc 96-Well Polypropylene DeepWell Storage Plate, Thermo Scientific, Cat.: 278743). High-throughput DNA extraction was carried out on an automated liquid handling platform (Microlab STAR Liquid Handling System, Hamilton, Cat.: Microlab STAR) using the ZymoBIOMICS 96 MagBead DNA Kit (Zymo Research, Cat.: D4308) on the Hamilton Star according to the manufacturer’s instructions. Purified samples were eluted into 50*μ*l ZymoBIOMICS DNase/RNase Free Water.

##### DNA extraction using an automated platform

The Maxwell RSC was used as a high throughput means of processing samples that used either the Copan or Isohelix swab collection method. To process the Copan swab samples, 300*μ*l of Promega Maxwell Lysis buffer and 30*μ*l of Promega Maxwell Proteinase K was added to each collection tube, then allowed to incubate in a water bath at 54C for 20 min. Following lysis, Copan swab heads were cut off their stem using sterile scissors and transferred into a filter tube (ClickFit Microtube, Promega, Cat.: V4745). The filter containing the swab was placed into a 2ml Eppendorf tube and spun down at full speed for 2min. This step is necessary since the Copan swab material consists of a foam, which harbors the main liquid containing the extracted DNA. Next, the eluate was combined with the corresponding sample tube media and added to a well of the Maxprep cartridge (Maxwell RSC Buccal Swab Kit, Promega, Cat.: AS1640). Cartridges were processed using the Maxwell RSC Instrument following the manufacturer’s default instructions. Extracted DNA was eluted in 50*μ*l Promega Elution Buffer and stored at −80C.

To process the Isohelix swabs, 300*μ*l of Promega Maxwell Lysis buffer was added to each matrix tube before vortexing at full speed for 1 min. The Isohelix swab head material is non-porous, which allows for easy collection of the lysate. The total lysate from each matrix tube was moved to the added to a well of the Maxprep cartridge using a 3cc syringe syringe (Blunt fill needle with Luer-Lok tip 18-G x 1 1/2-in 3-mL syringe, BD, Cat.: 305060). The Maxwell RSC Instrument was run using the ‘Blood’ program according to manufacturer’s instructions. Samples were subsequently eluted in 50*μ*l Promega Elution Buffer and stored at −80C.

##### Library preparation and sequencing

Following DNA extraction, library preparation for Illumina NGS platforms was performed at HudsonAlpha Genome Center using the QIAGEN Gene Reader DNA Library Prep Kit I (QIAGEN, Cat.: 180435) as was previously described in Afshinnekoo et al. (2015). Briefly, this involved fragmenting with an LE Series Covaris sonicator (Woburn, MA) with a targeted average size of 500nt, a bead clean-up step to remove fragments under 200nt, A-tailing, adaptor ligation, PCR amplification, bead-based library size selection, and a final clean-up step. A BioAnalyzer 2100 (Agilent, Cat.: G2939BA) was used to ensure libraries fell within a range of 450-650bp. Pilot samples collected in Barcelona and Stockholm were prepared using the QIAGEN QIAseq FX DNA Library Kit. The resulting libraries were sequenced on an Illumina HiSeq X Ten System (Illumina Inc., San Diego, CA) at HudsonAlpha Genome Center (Huntsville, Alabama) using HiSeq X Reagent Kits according to the manufacturer’s instructions (https://www.illumina.com).

#### Quality control

##### Evaluation of sequence quality

We measured sequencing quality based on 5 metrics: number of reads obtained from a sample, GC content, Shannon’s entropy of *k*-mers, post PCR Qubit score, and recorded DNA concentration before PCR. The number of reads in each sample was counted both before and after quality control, we used the number of reads after quality control for our results though the difference was slight. GC content was estimated from 100,000 reads in each sample after low quality DNA and human reads had been removed. Shannon’s entropy of *k*-mers was estimated from 10,000 reads taken from each samples. PCR Qubit score and DNA concentration are described in the wet lab methods.

We observed good separation of negative and positive controls based on both PCR Qubit and *k*-mer entropy. Distributions of DNA concentration and the number of reads were as expected (Figure S2G, H, I). GC content was broadly distributed for negative controls while positive controls were tightly clustered, expected since positive controls have a consistent taxonomic profile. Comparing the number of reads before and after quality control did not reveal any major outliers.

##### Identification of potential batch effects

Batch effects are a major concern for this low-biomass study and any large-scale study. The median flowcell used in our study contained samples from 3 cities and 2 continents. However, two flowcells covered 18 cities from 5 or 6 continents respectively. When samples from these flowcells were plotted using UMAP (see global diversity varies according to key covariates for details) the major global trends we described were recapitulated (Figure S2F). Plots of the number of reads against region (Figure S2G) showed a stable distribution of reads across cities. Analogous plots of PCR Qubit scores were less stable than the number of reads but showed a clear drop for control samples (Figure S2H). These results led us to conclude that batch effects are likely to be minimal.

##### Identification of potential strain contamination

We used BLASTn to align nucelotide assemblies from case samples to control samples. We used a threshold of 8,000 base pairs and 99.99% identity as a minimum to consider two sequences homologous. This threshold was chosen to be sensitive without solely capturing conserved regions. We identified all connected groups of homologous sequences and found approximate taxonomic identifications by aligning contigs to NCBI-NT using BLASTn searching for 90% nucleotide identity over half the length of the longest contig in each group.

Despite good separation of positive and negative controls (see STAR Methods) we identified several species in our negative controls which were also identified as prominent taxa in the data-set as a whole (See a core urban microbiome centers global diversity). Our dilemma was that a microbial species that is common in the urban environment might also reasonably be expected to be common in the lab environment. In general, negative controls had lower k-mer complexity, fewer reads, and lower post PCR Qubit scores than case samples and no major flowcell specific species were observed. Similarly, positive control samples were not heavily contaminated. These results suggest samples are high quality but do not systematically exclude the possibility of contamination.

Previous studies have reported that microbial species whose relative abundance is negatively correlated with DNA concentration may be contaminants. We observed a number of species that were negatively correlated with DNA concentration but this distribution followed the same shape as a null distribution of uniformly randomly generated relative abundances leading us to conclude that negative correlation may simply be a statistical artifact.

We analyzed the total complexity of case samples in comparison to control samples. Case samples had a significantly higher taxonomic diversity (Figure S2I) than any type of negative control sample. We also compared the confidence of taxonomic assignments to control assignments for prominent taxa using the number of unique marker *k*-mers to compare assignments. We found that case samples had more and higher quality assignments than could be found in controls. In contrast, the taxonomic assignment of one species, *Bradyrhizobium sp. BTAi1*, was not clearly more accurate in case samples than controls. Nevertheless, we were able to assemble genomes for this species in several unique samples, so we feel the species is not definitively a negative control contaminant.

Finally, we compared assemblies from negative controls to assemblies from our case samples searching for regions of high similarity that could be from an identical microbial strain. We reasoned that uncontaminated samples may contain the same species as negative controls but were less likely to contain identical strains. Only 137 case samples were observed to have any sequence with high similarity to an assembled sequence from a negative control (8,000 base pairs minimum of 99.99% identity). The identified sequences were principally from *Bradyrhizobium* and *Cutibacterium*. Since these genera are core taxa (See a core urban microbiome centers global diversity) observed in nearly every sample but high similarity was only identified in a few samples, we elected not to remove species from these genera from case samples.

##### Comparison of taxonomic and *k*-mer based metrics to establish database quality

We generated 31-mer profiles for raw reads using Jellyfish. All *k*-mers that occurred at least twice in a given sample were retained. We also generated MASH sketches from the non-human reads of each sample with 10 million unique minimizers per sketch. We calculated the Shannon’s entropy of *k*-mers by sampling 31-mers from a uniform 10,000 reads per sample.

We found clear correlations between *k*-mer based Jaccard distance (MASH) and taxonomic Jaccard distance (Figure S2A). We also compared alpha diversity metrics (Figure S2B): Shannon entropy of *k*-mers, and Shannon entropy of taxonomic profiles. As with pairwise distances these metrics were correlated though noise was present. This noise may reflect sub-species taxonomic variation in our samples.

##### Evaluation of unmapped DNA to establish aligner performance

A large proportion of the reads in our samples were not mapped to any reference sequence. There are three major reasons why a fragment of DNA would not be classified in our analysis 1) The DNA originated from a non-human and non-microbial species which would not be present in the databases we used for classification 2) Our classifier (KrakenUniq) failed to classify a DNA fragment that was in the database due to slight mismatch 3) The DNA fragment is not represented in any existing database. Explanations (1) and (2) are essentially drawbacks of the database and computational model used, and we can quantify them by mapping reads using a more sensitive aligner to a larger database, such as BLASTn (Altschul et al., 1990), or ensemble methods for analysis (McIntyre et al., 2017). To estimate the proportion of reads which could be assigned, we took 10k read subsets from each sample and mapped these to a set of large database using BLASTn (see a core urban microbiome centers global diversity for details). This resulted in 34.6% reads which could not be mapped to any external database compared to 41.3% of reads mapped using our approach with KrakenUniq. We note that our approach to estimate the fraction of reads that could be classified using BLASTn does not account for hits to low quality taxa which would ultimately be discarded in our pipeline, and so represents a worst-case comparison. Explanation (3) is altogether more interesting and we refer to this DNA as true unclassified DNA. In this analysis we do not seek to quantify the origins of true unclassified DNA except to postulate that it may derive from previously unknown species as have been identified in other similar studies.

#### Computational analysis of sequencing data

We processed raw reads from all samples into taxonomic, functional and AMR profiles for each sample using the MetaSUB Core Analysis Pipeline (Danko and Mason, 2020) (v1.0.0). This pipeline includes a preprocessing stage followed by steps to evaluate the taxonomic, functional, and *k*-mer profiles of metagenomic samples.

##### Sequence preprocessing

Sequence data were processed with AdapterRemoval (v2.17, Schubert et al. (2016)) to remove low quality reads and reads with ambiguous bases. Subsequently reads were aligned to the human genome (hg38, including alternate contigs) using Bowtie2 (v2.3.0, fast preset, Langmead and Salzberg (2013)). Read pairs where both ends mapped to the human genome were separated from read pairs where neither mate mapped. Read pairs where only one mate mapped were discarded. Hereafter, we refer to the read sets as human reads and non-human reads.

##### Generating taxonomic profiles for samples

We generated taxonomic profiles by processing non-human reads with KrakenUniq (v0.3.2 Breitwieser et al. (2018)) using a database based on all draft and reference genomes in NCBI/RefSeq Microbial (bacteria/archaea, fungi and virus) ca. March 2017. KrakenUniq was selected because its high performance, as it has been demonstrated to be comparable or having higher sensitivity than the best tools identified in a recent benchmarking study (McIntyre et al. (2017)) on the same comparative dataset. In addition, KrakenUniq allows for tunable specificity and identifies *k*-mers that are unique to particular taxa in a database. Reads are broken into *k*-mers and searched against this database. Finally, the taxonomic makeup of a sample is given by identifying the taxa with the greatest leaf to ancestor weight.

KrakenUniq reports the number of unique marker *k*-mers assigned to each taxon, as well as the total number of reads, the fraction of available marker *k*-mers found, and the mean copy number of those *k*-mers. We found that requiring more *k*-mers to identify a species resulted in a roughly log-linear decrease in the total number of species identified without a plateau or any other clear point to set a threshold (Figure S2C).

At a minimum, for an initial taxonomic call, we required three reads assigned to a taxa with 64 unique marker *k*-mers. This setting captures a group of taxa with low abundance but reasonable (10%–20%) coverage of the *k*-mers in their marker set (Figure S2E). However, this also allows for a number of taxa with very high (105) duplication of the identified marker *k*-mers and very few *k*-mers per read which we believe is biologically implausible. To remove these we filtered taxonomic calls further by requiring that the number of reads not exceed ^10^ times the number of unique *k*-mers, unless the set of unique *k*-mers was saturated (*>* 90% completeness). We include a full list of all taxonomic calls from all samples including diagnostic values for each call. We do not attempt to classify reads below the species level in this study.

##### Evaluating taxonomic calls

We further evaluated prominent taxonomic classifications for sequence complexity and genome coverage. For each microbe evaluated we calculated two indices generated using a random subset of 152 samples: the average topological entropy of reads assigned to the microbe and the Gini-coefficient of read positions on the microbial genome. For brevity we refer to these as *mean sequence entropy* (MSE) and *coverage equality* (CE). The formula for topological entropy of a DNA sequence is described by Koslicki (2011). Values close to 0 correspond to low-complexity sequences and values near 1 are high complexity. In this work we use a word size of 3 with an overall sequence length of 64 since this readily fits into our reads. To find the MSE of a microbial classification we take the arithmetic mean of the topological entropy of all reads that map to a given microbial genome in a sample. The Gini-coefficient is a classic economic measure of income inequality. We repurpose it here to evaluate the evenness of read coverage over a microbial classification. Reads mapping to a microbial genome are assigned to a contiguous 10kbp bin and the Gini-coefficient of all bins is calculated. Like MSE, the Gini-coefficient is bounded in [0, 1]. Lower values indicate greater inequality, very low values indicate that a taxon may be misidentified from conserved and near conserved regions. We downloaded one representative genome per species evaluated and mapped all reads from samples to using Bowtie2 (sensitive-local preset). Indices were processed from alignments using a custom script. Species classifications with an average MSE less than 0.75 or CE less than 0.1 were flagged.

##### Estimating relative abundance of taxa

To determine relative abundance of taxa (where applicable) in each profile we sub-sampled each sample to 100,000 classified reads, computed the proportion of reads assigned to each taxon, and took the distribution of values from all samples. This was the minimum number of reads sufficient to maintain taxonomic richness (Figure S2D). We chose sub-sampling (sometimes referred to as rarefaction in the literature) based on the study by Weiss et al. (2017), showing that sub-sampling effectively estimates relative abundance. Note that we use the term prevalence to describe the fraction of samples where a given taxon is found at any abundance and we use the term relative abundance to describe the fraction of DNA in a sample from a given taxon.

##### Contextualizing samples

We compared our samples to metagenomic samples from the Human Microbiome Project and a metagenomic study of European soil samples using MASH (Ondov et al., 2016), a fast *k*-mer based comparison tool. We built MASH sketches from all samples with 10 million unique *k*-mers to ensure a sensitive and accurate comparison. We used MASH’s built-in Jaccard distance function to generate distances between our samples and HMP samples. We then took the distribution of distances to soil and to each particular human commensal community as a proxy for the actual similarity of our samples to the site.

We used the Microbe Directory (Shaaban et al., 2018) to annotate taxonomic calls. The Microbe Directory is a hand curated, machine readable, database of functional annotations for 5,000 microbial species.

##### Functional and metabolic analysis of samples

We analyzed the metabolic functions in each of our samples by processing non-human reads with HUMAnN2 (Franzosa et al., 2018). We aligned all reads to UniRef90 (Suzek et al., 2015) using DIAMOND (v0.8.36, (Buchfink et al., 2015)) and used HUMAnN2 to produce estimate of pathway abundance and completeness. We filtered all pathways that were less than 50% covered in a given sample but otherwise took the reported pathway abundance as is after relative abundance normalization (using HUMAnN2’s attached script).

High level categories of functional pathways were found by grouping positively correlated pathways and manually annotating resulting clusters.

##### Analysis of Antimicrobial Resistance Genes

We generated profiles of antimicrobial resistance genes using MegaRes (v1.0.1, Lakin et al. (2017)). To generate profiles from MegaRes, we mapped non-human reads to the MegaRes database using Bowtie2 (v2.3.0, very-sensitive presets, Langmead and Salzberg (2013)). Subsequently, alignments were analyzed using ResistomeAnalyzer (commit 15a52dd https://github.com/cdeanj/resistomeanalyzer) and normalized by total reads per sample and gene length to give RPKMs. MegaRes includes an ontology grouping resistance genes into gene classes, AMR mechanisms, and gene groups. AMR detection remains a difficult problem and we note that detection of a homologous sequence to a known AMR gene does not necessarily imply an equivalent resistance in our samples. Currently, the gold standard for detecting AMR is via culturing.

Known AMR genes can come from gene families with homologous regions of sequence. To reduce spurious mapping from gene homology we used BLASTn to align all MegaRes AMR genes against themselves. We considered any connected group of genes with an average nucleotide identity of 80% across 50% of the gene length as a set of potentially confounded genes. We collapsed all such groups into a single pseudo-gene with the mean abundance of all constituent genes. Before clustering genes we removed all genes which were annotated as requiring SNP verification to predict resistance.

##### Analysis of Alpha and Beta Diversity

Inter-sample (beta) diversity was measured by the Jaccard distances between the taxonomic and functional profiles of samples. Jaccard distance does not use relative abundance information. Matrices of Jaccard distances were produced using built in SciPy (Virtanen et al., 2020) functions treating all elements greater than 0 as present. Hierarchical clustering (average linkage) was performed on the matrix of Jaccard distances using SciPy.

Dimensionality reduction of taxonomic and functional profiles was performed using UMAP (McInnes et al., 2018) on the matrix of Jaccard distances with 100 neighbors (UMAP-learn package, random seed of 42). We did not use Principal Component Analysis as a preprocessing step before UMAP as is sometimes done for high dimensional data.

Intra-sample (alpha) diversity was measured by using Species Richness and Shannon’s Entropy. We took species richness as the total number of detected species in a sample after rarefaction to 1 million reads. Shannon’s entropy is defined as *H* = *a*_*i*_*log*_2_*a*_*i*_ where *a*_*i*_ is the relative abundance of taxon *i* in the sample. This formulation is robust to sample read depth and accounts for the relative size of each group in diversity estimation. For alpha diversity based on *k*-mers or pathways, we simply substitute the relative abundance of a species for the relative abundance of the relevant type of object.

#### Identifying bacteria and archaea

##### Metagenomic assembly and binning

All samples were assembled with metaSPAdes (v3.10.1 Nurk et al. (2017)) using the *Bridges* system at the Pittsburgh Supercomputing Center (PSC) available through the Extreme Science and Engineering Discovery Environment (XSEDE) (Nystrom et al., 2015; Towns et al., 2014); contigs with length < 1000nt were excluded from further analysis. We mapped reads back to the remaining contigs via Bowtie2 (v2.3.4 Langmead and Salzberg (2013)) using the –very-sensitive-local preset to generate coverage metrics for each contig. Contigs with coverage information were binned using MetaBAT2 (v2.12.1 Kang et al. (2019)) with default parameters, resulting in 14,080 bins. Draft genome quality of each bin was assessed via CheckM (v1.0.13 Parks et al. (2015)) using the lineage_wf workflow with default parameters. Using the strategy proposed by Parks et al. (2018) we filtered bins by quality score, defined as QS = completeness - 5 ^∗^ contamination; bins with QS < 50 were removed from consideration. The remaining 6,107 bins were labeled by quality based on the MIMAG standard (Bowers et al. (2018)), with minor modification: 1,448 high quality (completeness > 90%, contamination < 5%, strain heterogeneity < 0.5%) bins, 4,532 medium quality (completeness > 50%, contamination < 5%) bins, all others low quality. Bins of at least medium quality were selected as acceptable Metagenome Assembled Genomes (MAGs) (5,980 total). PSC *Bridges* and XSEDE were used in the processing of these assemblies (Nystrom et al. (2015), Towns et al. (2014)).

##### Identifying replicated MAGs

OTUs (representative MAGs from a cluster) were chosen with a two-step clustering strategy. Rough single-linkage clustering formed primary clusters of MAGs based on Mash ANI (v2.1.1), with intra-cluster identity at 90%. Though Mash ANI can be inaccurate for potentially incomplete genomes (Olm et al. (2017)), we can leverage the technique’s speed for the many pairwise comparisons needed in this granular step. Within primary clusters, MAGs were compared pairwise by a more accurate whole-genome ANI (gANI) via dnadiff (v1.3) from MUMmer (v3.23 Kurtz et al. (2004)). Secondary, more refined clusters were grouped based n gANI using average-linkage hierarchical clustering from the R package dendextend (v1.12.0 Galili (2015)). A gANI cut-off of 95% resulted in 1,304 representative OTUs.

##### Matching OTUs to Reference Genomes

OTUs were compared against reference genomes from RefSeq (release 96 from November 2019, complete bacterial and archaeal genomes only, with “Exclude anomalous” and “Exclude derived from surveillance project” applied) as well as the full Integrated Gut Genomes (IGG) dataset (v1.0 Nayfach et al. (2019); 23,790 representative genomes). A MinHash sketch was created for each reference genome via Mash (v2.1.1) with default parameters to find Mash distances and select candidate “best matches” from each reference database. Then, dnadiff (v1.3) was used to further quantify differences between each OTU and its best match from either database. ANI between OTUs and their matches was found as “M-to-M AvgIdentity” in the query report column (ANI 95% over 60% OTU sequence qualified as a match).

##### OTU Taxonomic Assignment

OTUs were placed into a bacterial or archaeal reference tree (based on the Genome Database Taxonomy, GTDB Parks et al. (2020)) and then assigned taxonomic classifications using GTDB-Tk (v1.0.2 Chaumeil et al. (2019)). GTDB-Tk relies on 120 bacterial and 122 archaeal marker genes; domain assignment is chosen based on domain-specific marker content of the OTU sequence. Using the GTDB-Tk placements, we built an OTU-only bacterial phylogeny with FastTree (v2.1.10 Price et al. (2010)). The tree was visualized using iTOL (v5.5 Letunic and Bork (2019)).

##### Viral Discovery

We followed the protocol described by Paez-Espino et al. (2017). Briefly, we used an expanded and curated set of viral protein families (VPFs) as bait in combination with recommended filtering steps to identify 16,584 UViGs directly from all MetaSUB metagenomic assemblies greater than 5kb. Then, the UViGs were clustered with the content of the IMG/VR system (a total of over 730k viral sequences including isolate viruses, prophages, and UViGs from all kind of habitats). The clustering step relied on a sequence-based classification framework (based on 95% sequence identity across 85% of the shortest sequence length) followed by the markov clustering (mcl). This approach yielded 2,009 viral clusters (ranging from 2-611 members) and 9,605 singletons (or viral clusters of 1 member), sequences that failed to cluster with any sequence from the dataset or the references from IMG/VR, resulting in a total of 11,614 vOTUs. We define viral species from vOTUs as sequences sharing at least 95% identity over 85% of their length. Out of this total MetaSUB viral diversity, only 686 vOTUs clustered with any known viral sequence in IMG/VR.

##### Identifying Host Virus Interactions

We used two computational methods to reveal putative host-virus connections (Paez-Espino et al., 2016a). (1) For the 686 vOTUs that clustered with viral sequences from the IMG/VR system, we projected the known host information to all the members of the group (total of 2,064 MetaSUB UViGs). (2) We used bacterial/archaeal CRISPR-Cas spacer matches (from the IMG/M 1.1 million isolate spacer database) to the UViGs (allowing only for 1 SNP over the whole spacer length) to assigned a host to 1,915 MetaSUB vOTUs. Additionally, we also used a database of over 20 million CRISPR-Cas spacers identified from metagenomic contigs from the IMG/M system with taxonomy assigned. Since some of these spacers may derive from short contigs these results should be interpreted with caution.

##### CRISPR Array Detection and Annotation

Using CRISPRCasFinder (Couvin et al., 2018) the MetaSUB database was investigated to predict CRISPR arrays and annotate them with their corresponding predicted type based on CRISPR-Cas genes in their vicinity. CRISPRCasFinder was run with default parameters, “-so” and “-cas” options to identify cas genes. The precision and recall of the virus detection was 99.6% and 37.5% respectively, as previously reported by (Paez-Espino et al., 2016).

CRISPR-Cas types were assigned to arrays based on detected cas genes within a 10 kilobases vicinity. Cases where CRISPRCasFinder associated several cas genes of contradicting CRISPR-Cas types with the same CRISPR array were regarded as unclear annotation. This procedure yielded 838,532 predicted CRISPR arrays (with additional CRISPR arrays predicted with default parameters for PILER-CR), of which, 3,245 CRISPR arrays had unambiguous annotation, resulting in 43,656 unique spacers queried against genomic databases using BLASTN.

##### Matching CRISPR Spacers to Organism Databases

In order to associate detected spacers within defined groups (plasmids, prophages, viruses) four different genomic databases were aggregated to be searched with BLASTN. The aggregated database consisted of IMG/VR, PHASTER, and PLSDB alongside bacterial and archaeal genomic sequences from the National Center for Biotechnology Information (NCBI). All database downloads were made on the 28th January 2020. Detected and annotated spacers were searched against the databases mentioned above using BLASTN with the following additional arguments, which correspond to the default parameters of CRISPRTarget: word_size = 7, evalue = 1, gapopen = 10, gapextend = 2, penalty = −1, reward = 1.

#### GeoDNA Sequence Search

For building the sequence graph index, each sample was processed with KMC (version 3, Kokot et al., 2017) to convert the reads in FASTA format into lists of *k*-mer counts, using different values of *k* ranging from 13 to 19 in increments of 2. All *k*-mers that contained the character “N” or occurred in a sample less than twice were removed. For each value of *k*, we built a separate index, consisting of a labeled de Bruijn graph, using an implicit representation of the complete graph and a compressed label representation based on Multiary Binary Relation Wavelet Trees (Multi-BRWT). For further details, we refer to the manuscript (Karasikov et al., 2020). To build the index, for each sample the KMC *k*-mer count lists were transformed into de Bruijn graphs, from which path covers in the form of contig sets were extracted and stored as intermediate FASTA files. The contig sets of each sample were then transformed into annotation columns (one column per sample) by mapping them onto an implicit complete de Bruijn graph of order *k*. All annotation columns were then merged into a joint annotation matrix and transformed into Multi-BRWT format. Finally, the topology of the Multi-BRWT representation was optimized by relaxing its internal tree arity constraints to allow for a maximum arity of 40.

### Quantification and statistical analysis

For each statistical test in this manuscript, the type of test, the size (n) of the test, and statistical summaries or measures of dispersion are clearly defined in the figure legends or in the accompanying text throughout the manuscript.

### Additional resources

#### Interactive visualizations and maps

https://pngb.io/metasub-maps

#### BLAST-like sequence search tool

https://dnaloc.ethz.ch

#### Raw and Analyzed Data Files

https://pngb.io/metasub-2021

#### Collated Metadata

https://pngb.io/metasub-2021, https://github.com/MetaSUB/MetaSUB-metadata

#### Jupyter notebooks used to generate the figures and statistics in this study

https://www.github.com/MetaSUB/main_paper_figures

## Supplemental information

Document S1. Tables S1 and S2

Video S1. MetaSUB sampling protocol, related to STAR Methods and Table 1
