# Supplementary material for: A global metagenomic map of urban microbiomes and antimicrobial resistance
Source: Cell. 2021 Jun 24;184(13):3376–3393.e17. doi: 10.1016/j.cell.2021.05.002 (PMC8238498; doi:10.1016/j.cell.2021.05.002)
Supplement: Document S1. Tables S1 and S2 [file mmc1.pdf]

Cell, Volume *184*

Supplemental information

A global metagenomic map of urban microbiomes and antimicrobial resistance

David Danko, Daniela Bezdan, Evan E. Afshin, Sofia Ahsanuddin, Chandrima Bhattacharya, Daniel J. Butler, Kern Rei Chng, Daisy Donnellan, Jochen Hecht, Katelyn Jackson, Katerina Kuchin, Mikhail Karasikov, Abigail Lyons, Lauren Mak, Dmitry Meleshko, Harun Mustafa, Beth Mutai, Russell Y. Neches, Amanda Ng, Olga Nikolayeva, Tatyana Nikolayeva, Eileen Png, Krista A. Ryon, Jorge L. Sanchez, Heba Shaaban, Maria A. Sierra, Dominique Thomas, Ben Young, Omar O. Abudayyeh, Josue Alicea, Malay Bhattacharyya, Ran Blekhman, Eduardo Castro-Nallar, Ana M. Cañas, Aspassia D. Chatziefthimiou, Robert W. Crawford, Francesca De Filippis, Youping Deng, Christelle Desnues, Emmanuel Dias-Neto, Marius Dybwad, Eran Elhaik, Danilo Ercolini, Alina Frolova, Dennis Gankin, Jonathan S. Gootenberg, Alexandra B. Graf, David C. Green, Iman Hajirasouliha, Jaden J.A. Hastings, Mark Hernandez, Gregorio Iraola, Soojin Jang, Andre Kahles, Frank J. Kelly, Kaymisha Knights, Nikos C. Kyrpides, Paweł P. Łabaj, Patrick K.H. Lee, Marcus H.Y. Leung, Per O. Ljungdahl, Gabriella Mason-Buck, Ken McGrath, Cem Meydan, Emmanuel F. Mongodin, Milton Ozorio Moraes, Niranjan Nagarajan, Marina Nieto-Caballero, Houtan Noushmehr, Manuela Oliveira, Stephan Ossowski, Olayinka O. Osuolale, Orhan Özcan, David Paez-Espino, Nicolás Rascovan, Hugues Richard, Gunnar Rätsch, Lynn M. Schriml, Torsten Semmler, Osman U. Sezerman, Leming Shi, Tieliu Shi, Rania Siam, Le Huu Song, Haruo Suzuki, Denise Syndercombe Court, Scott W. Tighe, Xinzhao Tong, Klas I. Udekwu, Juan A. Ugalde, Brandon Valentine, Dimitar I. Vassilev, Elena M. Vayndorf, Thirumalaisamy P. Velavan, Jun Wu, María M. Zambrano, Jifeng Zhu, Sibo Zhu, Christopher E. Mason, and The International MetaSUB Consortium

Table S1: Sample Counts by City, Related to Table 1.

Region	city	Pilot	CSD16	CSD17	Other	Total
East Asia	Region Total	34.0	26.0	1300	0	1357
	Fukuoka	0.0	0.0	3	0	158
	Hanoi	0.0	0.0	16	0	16
	Hong Kong	12.0	0.0	712	0	724
	Kuala Lumpur	0.0	0.0	30	0	30
	Sendai	0.0	0.0	32	0	32
	Seoul	12.0	0.0	80	0	92
	Shanghai	10.0	0.0	0	0	10
	Singapore	0.0	0.0	192	0	192
	Taipei	0.0	0.0	94	0	94
	Tokyo	0.0	26.0	132	0	158
	Yamaguchi	0.0	0.0	9	0	9
Europe	Region Total	177.0	310.0	939	1	1427
	Barcelona	25.0	99.0	0	0	124
	Belfast	0.0	0.0	5	0	5
	Berlin	0.0	55.0	1	0	56
	Birmingham	0.0	0.0	5	1	6
	Bradford	0.0	0.0	4	0	4
	Bury	0.0	0.0	6	0	6
	Eastbourne	0.0	0.0	6	0	6
	Eden	0.0	0.0	5	0	5
	Edinburgh	0.0	0.0	6	0	6
	Islington	0.0	0.0	5	0	5
	Jaywick	0.0	0.0	6	0	6
	Kensington	0.0	0.0	6	0	6
	Kyiv	0.0	0.0	97	0	97
	Lands End	0.0	0.0	5	0	5
	Lisbon	28.0	60.0	0	0	88
	London	0.0	0.0	534	0	534
	Marseille	0.0	96.0	16	0	112
	Naples	0.0	0.0	16	0	16
	Newcastle	0.0	0.0	5	0	5
	Oslo	12.0	0.0	16	0	28
	Paris	0.0	0.0	16	0	16
	Porto	112.0	0.0	0	0	112
	Sofia	0.0	0.0	16	0	16
	Stockholm	0.0	0.0	62	0	62
	Swansea	0.0	0.0	6	0	6
	Vienna	0.0	0.0	16	0	16
	Zurich	0.0	0.0	79	0	79
Middle East	Region Total	0.0	100.0	15	0	115
	Doha	0.0	100.0	15	0	115

continent	project city	Pilot	CSD16	CSD17	Other	Total
North America	Region Total	28.0	284.0	371	276	959
	Baltimore	0.0	0.0	23	0	23
	Denver	0.0	24.0	23	0	47
	Fairbanks	0.0	141.0	0	0	141
	Mexico City	10.0	0.0	0	0	10
	Minneapolis	0.0	0.0	16	0	16
	New York City	0.0	103.0	279	276	658
	Sacramento	18.0	16.0	0	0	34
	San Francisco	0.0	0.0	30	0	30
Oceania	Region Total	0.0	94.0	32	0	126
	Auckland	0.0	16.0	0	0	16
	Brisbane	0.0	0.0	16	0	16
	Hamilton	0.0	16.0	0	0	16
	Honolulu	0.0	0.0	16	0	16
	Sydney	0.0	62.0	0	0	62
South America	Region Total	20.0	44.0	199	68	331
	Bogota	0.0	17.0	0	0	17
	Montevideo	20.0	0.0	0	0	20
	Ribeirao Preto	0.0	0.0	93	0	93
	Rio De Janeiro	0.0	0.0	77	68	145
	Santiago	0.0	27.0	0	0	27
	Sao Paulo	0.0	0.0	29	0	29
Sub Saharan Africa	Region Total	0.0	116.0	192	0	308
	Ilorin	0.0	90.0	134	0	224
	Offa	0.0	26.0	58	0	84

Table S2: Covariate Variance, Related to Figure 2. The sample variance that can be explained by each factor, in isolation.

Factor	Variance Explained
City	19%
City Population Density	0%
City Ave June Temp	4%
City Elevation	2%
Coastal City	1%
Surface Material	4%
Koppen Climate Classification	8%
Setting	2%
Above/Below Ground	7%
Continent	11%
